# Mitochondrial Quality Control in Degenerative Musculoskeletal Diseases: Signaling Networks and Therapeutic Landscapes

**DOI:** 10.1002/advs.78309

**Published:** 2026-10-14

**Authors:** Haiyang Gao, Zhangrong Cheng, Xianglong Chen, Wenbo Wu, Wang Wu, Zimu Yu, Cao Yang, Yukun Zhang

**Affiliations:** ^1^ Department of Orthopaedics, Union Hospital, Tongji Medical College Huazhong University of Science and Technology Wuhan Hubei Province China

**Keywords:** degenerative musculoskeletal diseases, epigenetics, mitochondrial quality control, molecular subtyping, targeted therapy

## Abstract

Degenerative musculoskeletal disorders, including osteoarthritis (OA), intervertebral disc degeneration (IVDD), and osteoporosis (OP), are increasingly viewed as disorders of mitochondrial quality control (MQC) rather than passive mechanical wear. The MQC network, comprising mitochondrial dynamics, mitophagy‐biogenesis coupling, and inter‐organelle communication, organizes the aging‐inflammation‐metabolism axis. Its collapse releases mtDNA fragments that act as damage‐associated molecular patterns (DAMPs) activating innate immunity via cGAS‐STING, and metabolic intermediates that remodel the epigenome through histone lactylation, potentially locking acute stress into a “metabolic memory”, although the mtDNA‐methylation component remains hypothesis‐level. Multi‐omics and cell‐free mtDNA liquid biopsy underpin a proposed subtyping into bioenergetic failure, immuno‐inflammatory activation, and senescent cell accumulation, still a within‐OA hypothesis pending independent and cross‐disease validation. Therapy is shifting from symptom management toward organelle medicine, including cardiolipin stabilizers, PROTAC‐mediated protein degradation, mitochondrial base editing, and engineered mitochondrial transplantation. Metformin has randomized trial evidence in knee OA pending confirmation, NAD+ precursors are in early clinical testing, and SS‐31 holds accelerated approval only for Barth syndrome; other strategies remain preclinical. Delivery barriers and disease heterogeneity require precision stratification, biomimetic models, and AI‐driven decision‐making. This review delineates the shared molecular logic of the three diseases and proposes a prototype subtyping‐to‐treatment roadmap pending validation.

## Graphical abstract

Mitochondrial quality control (MQC) collapse redefines osteoarthritis, intervertebral disc degeneration, and osteoporosis as disorders of organelle dysfunction. Damaged mitochondria leak mtDNA and metabolites, driving cGAS‐STING inflammation, epigenetic reprogramming, and metabolic memory. Molecular subtyping enables biomarker‐guided targeting (from NAD^+^ precursors to STING inhibitors) shifting treatment from symptom palliation toward organelle restoration.

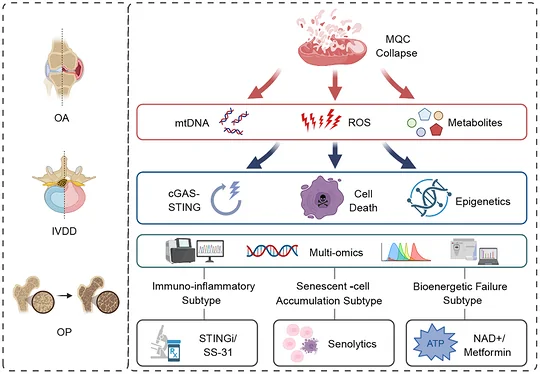

**Abbreviations**
2‐HG: 2‐hydroxyglutarate
5hmC: 5‐hydroxymethylcytosine
5Mc: 5‐methylcytosine
AI: artificial intelligence
AMP: adenosine monophosphate
AMPK: AMP‐activated protein kinase
ATP: adenosine triphosphate
BMD: bone mineral density
BoC: bone‐on‐a‐chip
CBP: CREB‐binding protein
ccf‐mtDNA: circulating cell‐free mitochondrial DNA
cGAMP: cyclic GMP‐AMP
cGAS: cyclic GMP‐AMP synthase
COX: cytochrome c oxidase
CRP: C‐reactive protein
DAMPs: damage‐associated molecular patterns
DdCBEs: DddA‐derived cytosine base editors
DMBADs: disease‐modifying bone anabolic drugs
DMOADs: disease‐modifying osteoarthritis drugs
Drp1: dynamin‐related protein 1
ECM: extracellular matrix
ER: endoplasmic reticulum
ESR: erythrocyte sedimentation rate
ETC: electron transport chain
GRP75: glucose‐regulated protein 75
HIF‐1α: hypoxia‐inducible factor 1α
IFN: interferon
IFN‐β: interferon‐β
IL: interleukin
IP3R: inositol 1,4,5‐trisphosphate receptor
IVDD: intervertebral disc degeneration
JoC: joint‐on‐a‐chip
KDM: histone demethylase
LC3‐II: microtubule‐associated protein 1 light chain 3‐II
LIPUS: low‐intensity pulsed ultrasound
LNP: lipid nanoparticle
MAMs: mitochondria‐associated membranes
MAPL/MUL1: mitochondrial E3 ubiquitin ligase
MFN1/2: mitofusin 1/2
MitoQ: mitoquinone mesylate
MMP: matrix metalloproteinase
MnSOD: manganese superoxide dismutase
mPTP: mitochondrial permeability transition pore
MQC: mitochondrial quality control
MRI: magnetic resonance imaging
MSC: mesenchymal stem cell
mtDNA: mitochondrial DNA
mTORC1: mechanistic target of rapamycin complex 1
NAD^+^
: nicotinamide adenine dinucleotide
NF‐κB: nuclear factor kappa‐B
NMN: nicotinamide mononucleotide
NR: nicotinamide riboside
NSAIDs: non‐steroidal anti‐inflammatory drugs
OA: osteoarthritis
OMM: outer mitochondrial membrane
OP: osteoporosis
OPA1: optic atrophy 1
OXPHOS: oxidative phosphorylation
PEMF: pulsed electromagnetic field
PGC‐1α: peroxisome proliferator‐activated receptor gamma coactivator 1α
PHDs: prolyl hydroxylases
PINK1: PTEN‐induced kinase 1
PROTACs: proteolysis‐targeting chimeras
PTMs: post‐translational modifications
RANKL: receptor activator of nuclear factor‐κB ligand
ROS: reactive oxygen species
SASP: senescence‐associated secretory phenotype
SA‐β‐Gal: senescence‐associated β‐galactosidase
SDH: succinate dehydrogenase
SENP5: sentrin/SUMO‐specific protease 5
SIRT3: sirtuin 3
SS‐31: elamipretide
STING: stimulator of interferon genes
SUMO: small ubiquitin‐like modifier
TCA: tricarboxylic acid
TET: ten‐eleven translocation enzyme
TFAM: mitochondrial transcription factor A
TFEB: transcription factor EB
TNF‐α: tumor necrosis factor alpha
TNT: tunneling nanotube
UGDH: UDP‐glucose dehydrogenase
UPR: unfolded protein response
VDAC1: voltage‐dependent anion channel 1
α‐KG: α‐ketoglutarate

## 1 Introduction

Degenerative musculoskeletal disorders, namely osteoarthritis (OA), intervertebral disc degeneration (IVDD), and osteoporosis (OP)‐were long attributed to passive “mechanical wear” and structural fatigue of articular cartilage, nucleus pulposus, and trabecular bone [1]. Aging and metabolic epidemics have exposed paradoxes that a purely biomechanical model cannot explain: patients with metabolic syndrome develop severe OA even in non‐weight‐bearing hand joints [2, 3], and low‐grade systemic inflammation also accelerates bone loss [4]. These observations have shifted disease concepts from macroscopic structural failure toward a framework of cellular metabolic and signaling network crisis [5], in which the musculoskeletal microenvironment is an active immuno‐metabolic signaling network whose core pathogenic driver is the mitochondrion. Mitochondria integrate mechanical [6] and inflammatory signals while fueling matrix synthesis; when they fail to adapt to external stress, tissue‐level failure of energy‐signal coupling ensues.

Although molecular understanding of disease mechanisms has reached the single‐cell level [7], clinical management has lagged: from NSAIDs and glucocorticoid injections to joint replacement or spinal fusion, current strategies remain essentially symptom management or structural salvage rather than disease modification [8, 9]. This gap underscores the urgent need for disease‐modifying drugs (DMOADs and DMBADs). Single‐target anti‐cytokine strategies have repeatedly failed in orthopedic trials: randomized controlled trials of canakinumab and etanercept did not meet pain or structural endpoints, and blocking one cytokine can be offset by compensatory activation of non‐canonical NF‐κB and JAK‐STAT pathways [8, 10]. This failure pattern suggests repositioning therapy upstream at the hub of the inflammatory network. Ddysregulation of the mitochondrial quality control (MQC) network constitutes precisely such a hub [11].

MQC is not a single biological event but an integrated signaling network encompassing mitochondrial biogenesis, dynamics (fusion and fission), mitophagy [12], and mitochondria‐ER interplay [13]. When MQC decompensates under aging, chronic inflammation, or metabolic overload, damaged mitochondria trigger a bioenergetic crisis while leaking mitochondrial DNA (mtDNA) and reactive oxygen species (ROS) into the cytosol and releasing metabolic intermediates such as succinate [14]. These leaked molecules act as damage‐associated molecular patterns (DAMPs) and retrograde signaling messengers that persistently activate innate immune pathways such as cGAS‐STING; through epigenetic mechanisms including histone lactylation, they may lock cells into “metabolic memory” and thereby sustain a vicious cycle of sterile inflammation and tissue degeneration.

Recent systematic reviews have summarized the central role of MQC in OA and other joint diseases, mtDNA/ROS‐driven cGAS‐STING inflammation, the functions of PGC‐1α, SIRT3, and PINK1/Parkin in bone metabolism, and mitochondria‐derived metabolite‐mediated epigenetic modifications [15, 16]. These reviews, however, have largely remained confined to single mechanisms or single diseases. This review instead integrates dispersed evidence into a conceptual cross‐disease framework organized around MQC: the aging‐inflammation‐metabolism axis converges on mitochondria, and MQC collapse may leave detectable molecular fingerprints (mtDNA leakage and metabolic memory) that link mechanism, subtyping, and treatment matching. Every link in this chain remains to be prospectively validated.

Accordingly, this review is organized along four interconnected dimensions. First, using MQC as the axis, we delineate the shared molecular logic and tissue‐specific divergence of OA, IVDD, and OP. Second, we trace how mitochondrial metabolic intermediates (lactate, succinate, and α‐KG/2‐HG) reshape the histone modification landscape, converting reversible acute stress into chronic “metabolic memory”. Third, in light of the clinical impasse of single‐target anti‐inflammatory strategies, we stratify MQC‐targeted interventions by evidence level. Fourth, we construct a preliminary MQC‐based subtyping framework interfacing with biomarker‐enriched trial design. Throughout, we distinguish findings that are established from those that remain emerging or controversial, and we avoid implying that a mechanism demonstrated in one tissue necessarily operates in the others. The resulting framework is integrative rather than definitive, and its clinical utility will depend on prospective validation of its central claims.

## 2 Mitochondrial Quality Control Network: Molecular Landscape and Signal Transduction Architecture

The mitochondrial quality control (MQC) system maintains cellular energy homeostasis by coordinating mitochondrial dynamics (fusion and fission), biogenesis, and clearance of damaged organelles [17]. Matrix synthesis by chondrocytes, mineralization by osteoblasts, and bone resorption by osteoclasts all depend on spatiotemporal control of energy metabolism by MQC. This section examines the three core dimensions of the MQC network‐mitochondrial dynamics, the mitophagy‐biogenesis cycle, and inter‐organelle communication, and their cooperative control of cell fate through post‐translational modifications (PTMs), kinase cascades, and protein machines (Figure 1).

**FIGURE 1:**
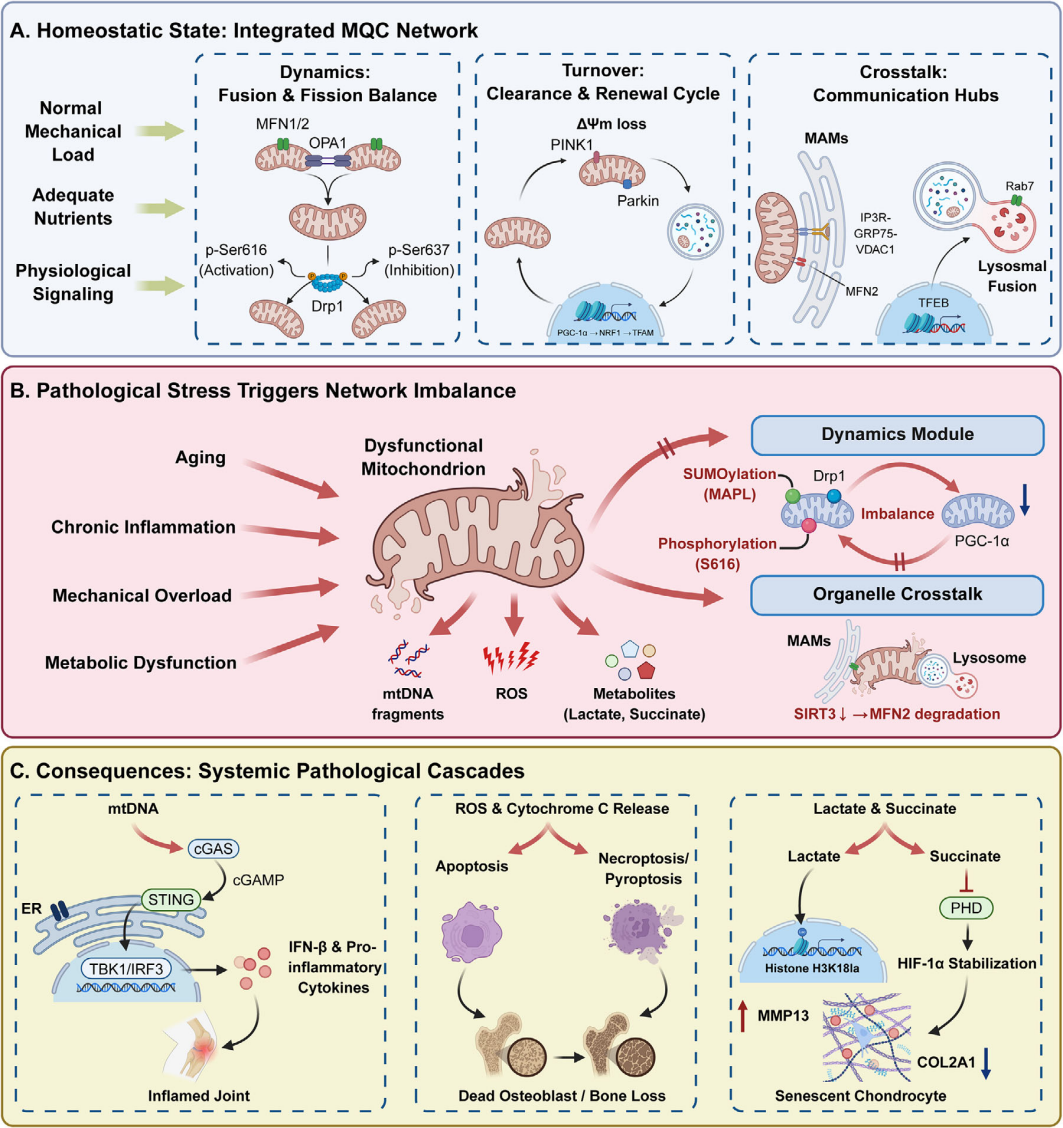
Central role of the MQC network in musculoskeletal homeostasis and disease. A healthy MQC network maintains cellular homeostasis, whereas its collapse under stress drives degenerative musculoskeletal diseases. (A, B) Homeostatic maintenance: under steady‐state inputs such as normal mechanical load, adequate nutrition, and youthful hormonal signals, functional mitochondria maintain homeostasis through three interlocked modules: dynamic balance (fusion‐fission cycling); the mitophagy‐biogenesis cycle (PINK1/Parkin‐mediated autophagic clearance coupled with PGC‐1α‐driven biogenesis); and organelle interplay (MAM contact sites with the ER and fusion with lysosomes). (C) Network collapse and pathogenic cascade: chronic stress causes MQC network dysfunction; damaged mitochondria release mtDNA fragments, ROS, and metabolites (lactate, succinate), activating downstream pathogenic pathways‐cytosolic mtDNA triggers cGAS‐STING‐mediated innate immune activation; metabolite accumulation drives epigenetic reprogramming; and ROS with cytochrome c release induce cell death (apoptosis and pyroptosis). Together these molecular events contribute to the tissue phenotypes of OA, OP, and IVDD.

### 2.1 Dynamic Remodeling: The Post‐Translational Regulatory Network Governing Mitochondrial Fusion and Fission

Mitochondrial dynamics is a morphological adaptation mechanism by which cells respond to changing metabolic demands. Execution is mediated by dynamin‐related protein 1 (Drp1) and other members of the dynamin superfamily, with regulation provided by a network of post‐translational modifications (PTMs) including phosphorylation, ubiquitination, SUMOylation, and acetylation [18]. Upon fission initiation, cytosolic Drp1 is recruited to the outer mitochondrial membrane (OMM), where it interacts with receptor proteins such as Mff, MiD49/51, and Fis1, oligomerizes into ring‐like structures, and drives membrane scission through GTP hydrolysis [19].

Phosphorylation is the core PTM controlling Drp1 activity. During osteoclast differentiation, RANKL triggers the MAPK/ERK cascade to phosphorylate Drp1 at Ser616, promoting its mitochondrial translocation and fission, which fragments mitochondria and upregulates glycolytic flux to support bone resorption metabolically [20]. In osteoblasts, PKA‐mediated phosphorylation at Ser637 inhibits Drp1 GTPase activity and impairs its OMM binding, favoring a fused mitochondrial network; this configuration is proposed to sustain the electron transport chain (ETC) output required for mineralization [20, 21].

SUMOylation constitutes another layer of fission regulation: the OMM E3 ligase MAPL (MUL1) catalyzes Drp1 SUMOylation, stabilizing its retention on the mitochondrial surface and acting as a molecular switch for pro‐apoptotic signaling under stress [22]. In intervertebral disc degeneration (IVDD), downregulation of the deSUMOylase SENP5 leads to hyperactivation of the MAPL‐Drp1 axis, driving extensive mitochondrial fragmentation in nucleus pulposus cells, inducing cell death and accelerating tissue degeneration [23].

Opposing fission, mitochondrial fusion is mediated by the OMM proteins mitofusin 1/2 (MFN1/2) and the inner membrane protein optic atrophy 1 (OPA1), buffering metabolic stress and mixing matrix contents through the formation of tubular networks [24]. MFN2 activity is dynamically regulated by acetylation: the mitochondrial deacetylase SIRT3 deacetylates MFN2, enhancing its GTPase activity and protecting it from ubiquitin‐proteasome degradation [25]. The loss of this protective SIRT3‐MFN2 axis in OA chondrocytes, and its impact on mitochondria‐ER contact site (MAMs) stability, are discussed in Section 3.2.2 in the context of organelle communication collapse.

These PTMs do not act in isolation but are interlinked through crosstalk: phosphorylation can influence the efficiency and functional outcome of subsequent SUMOylation and ubiquitination by altering substrate conformation or enzyme‐substrate accessibility [26]. An example of such crosstalk in skeletal disease is the SUMOylation steady‐state imbalance of MAPL‐Drp1 in intervertebral disc degeneration. The pathological significance of such crosstalk remains to be directly validated. One may nonetheless speculate that Ser616 phosphorylation superimposed on SUMOylation imbalance could synergistically drive osteoclast mitochondrial fragmentation and excessive bone resorption, whereas the protective effects of Ser637 phosphorylation may be counteracted by excessive SUMOylation. This hypothesis provides a framework for understanding the multi‐layered nature of MQC dysregulation and suggests that interventions targeting specific PTM combinations may outperform strategies aimed at single modifications.

### 2.2 Homeostatic Cycle: Coordinated Coupling of Mitophagy and Biogenesis

The MQC network depends on the dynamic coupling of mitophagy and biogenesis to continuously clear damaged mitochondria and replenish functional new units [27]. The clearance phase is executed by the PTEN‐induced kinase 1 (PINK1)/Parkin axis. In healthy mitochondria, PINK1 is constitutively imported into the inner membrane and cleaved by the protease PARL, maintaining low abundance [28]; upon depolarization of the mitochondrial membrane potential (ΔΨm), PINK1 import is blocked, and the kinase accumulates on the OMM, where dimerization and autophosphorylation activate it to phosphorylate ubiquitin and the ubiquitin‐like domain of the cytosolic E3 ubiquitin ligase Parkin, releasing its autoinhibition and recruiting it to damaged mitochondria [29]. Resident Parkin ubiquitinates OMM substrates including MFN2 and VDAC1, generating “eat‐me” signals recognized by autophagy receptors such as p62/SQSTM1 and OPTN, and the damaged mitochondria are ultimately degraded via the autophagy‐lysosomal pathway [30, 31].

Beyond marking mitochondria for mitophagy, Parkin‐mediated MFN2 ubiquitination also regulates the stability of mitochondria‐ER contact sites (MAMs), a non‐autophagic function that may be equally important in osteoblasts. Parkin regulates ER‐mitochondria contacts through Mfn2 [32], and its loss causes ER Ca^2+^ dyshomeostasis [33]; as an MAM anchoring protein, MFN2 ubiquitination status affects the maintenance of ER‐mitochondria contacts, indicating that the pathological effects of PINK1/Parkin deficiency cannot be attributed solely to reduced autophagic flux. In osteoblasts, this pathway is critical for clearing ROS‐damaged mitochondria generated by high metabolic activity; PINK1/Parkin deficiency exposes osteoblasts to persistent oxidative stress and triggers apoptosis [34]. The systemic consequences of PINK1/Parkin deficiency in bone loss and its relationship to energy metabolism competition are detailed in Section 3.4.1 in the context of osteoporosis.

Opposing clearance, the renewal phase is driven by the transcriptional coactivator peroxisome proliferator‐activated receptor γ coactivator 1α (PGC‐1α) [35]. Under energy depletion (elevated AMP/ATP ratio), AMPK phosphorylates and activates PGC‐1α, which is further deacetylated and activated by the NAD^+^‐dependent deacetylase SIRT1; activated PGC‐1α translocates to the nucleus and cooperates with nuclear respiratory factors (NRF1/2) to initiate transcription of nuclear‐encoded mitochondrial genes, including mitochondrial transcription factor A (TFAM), which drives mtDNA replication and transcription [36]. In OA and OP, suppression of PGC‐1α expression or activity generally precedes macroscopic tissue degeneration [37].

### 2.3 Key Inter‐Organelle Communication Networks

Beyond mitophagy and biogenesis, physical contacts and functional crosstalk between organelles constitute the third dimension of the MQC network; among these, mitochondria‐ER contact sites (MAMs) and mitochondria‐lysosome interactions are particularly critical in skeletal pathophysiology.

#### 2.3.1 Mitochondria‐ER Contact Sites: A Multidimensional Signaling Hub

The specialized membrane contact sites between mitochondria and the endoplasmic reticulum (ER), termed MAMs, are multidimensional signaling hubs that regulate cellular metabolism and fate [38]. The core complex is composed of the ER‐resident inositol 1,4,5‐trisphosphate receptor (IP3R), the adaptor protein glucose‐regulated protein 75 (GRP75), and the OMM voltage‐dependent anion channel 1 (VDAC1) [39], enabling direct and efficient transfer of ER Ca^2+^ into the mitochondrial matrix, where it activates key tricarboxylic acid (TCA) cycle dehydrogenases (pyruvate dehydrogenase PDH and α‐ketoglutarate dehydrogenase α‐KGDH) to support high‐energy‐demand processes such as osteoblast differentiation and bone formation. MAMs also serve as sites of lipid synthesis and intermembrane exchange: the ER‐resident protein VAPB and the mitochondrial protein PTPIP51 anchor each other, mediating phosphatidylserine (PS) transport to mitochondria and its conversion to phosphatidylethanolamine (PE), maintaining mitochondrial membrane integrity [40].

Under pathological conditions, this communication is readily perturbed: persistent overactivation of the IP3R‐GRP75‐VDAC1 channel can cause mitochondrial matrix Ca^2+^ overload, triggering opening of the mitochondrial permeability transition pore (mPTP), releasing cytochrome c and initiating the mitochondrial pathway of apoptosis; conversely, downregulation of MFN2, an MAM anchoring protein, causes dissociation of ER‐mitochondria contacts, blocking lipid exchange and Ca^2+^ signaling, and inducing ER stress and the unfolded protein response (UPR), accelerating chondrocyte senescence and apoptosis [41]. The specific pathological role of this mechanism in OA is detailed in Section 3.2.2.

#### 2.3.2 Mitochondria‐Lysosome Interplay: Terminal Regulation of Autophagic Flux

Completion of mitophagy depends on efficient fusion of autophagosomes with lysosomes and subsequent content degradation; this rate‐limiting step is regulated by mitochondria‐lysosome contacts [42]: the two organelles form dynamic physical contacts through specific membrane proteins and ion channels, mediating bidirectional exchange of lipids and Ca^2+^ to modulate the rhythm and efficiency of autophagic flux [43]. Transcription factor EB (TFEB) is the master regulator of the autophagy‐lysosomal system [44]. Under energy‐replete conditions, TFEB is phosphorylated by mTORC1 and retained in the cytosol; when mitochondrial damage triggers an energy crisis (elevated AMP/ATP ratio), AMPK suppresses mTORC1 and, together with ROS and local Ca^2+^ signals, promotes TFEB dephosphorylation and nuclear translocation, initiating transcription of target genes including lysosomal hydrolases, V‐ATPase, and autophagy receptors, thereby enhancing lysosomal biogenesis and degradation capacity [45].

In the pathological microenvironment of degenerative musculoskeletal diseases, this network is frequently severely dysregulated: in aging OA chondrocytes or osteoblasts under inflammatory stress, lysosomal acidification is impaired (elevated luminal pH), and the fusion‐related proteins Rab7 and STX17 are functionally compromised [46], so autophagosomes loaded with damaged mitochondria cannot fuse, leading to autophagosome accumulation. Rather than clearing mitochondrial debris, this ineffective autophagic flux consumes membrane resources and ATP, aggravating the metabolic burden [47]. Chronic pathological stress can also induce lysosomal membrane permeabilization (LMP), leaking hydrolases into the cytosol and driving skeletal cell apoptosis [48].

The three dimensions described above‐mitochondrial dynamics, the mitophagy‐biogenesis cycle, and inter‐organelle communication, are not independent but synergistically coupled, and the common consequence of their dysregulation is inflammation amplification driven by mtDNA/ROS leakage and failure of metabolic‐signaling coupling. At the dynamics level, fission and fusion are cross‐regulated by multiple PTMs, which provides a rationale for testing combination interventions rather than single‐site targeting. In the mitophagy‐biogenesis cycle, imbalance produces the dual consequences of insufficient clearance of damaged mitochondria and deficient supply of new units. The integrity of organelle contact sites is a major determinant of skeletal‐cell survival under stress, and its disintegration is a recurrent early event in degenerative pathology.

## 3 Core Mechanisms: Pathological Signaling Cascades and Network Collapse Driven by MQC Dysregulation

In the complex microenvironment of degenerative musculoskeletal diseases, MQC network collapse is far more than an isolated organelle dysfunction; it is a signaling cascade event that can propagate throughout the cell and the tissue microenvironment [49]. This chapter examines how, after MQC network collapse, mitochondria transform from homeostatic energy hubs into sources driving pathological cascades (Figure 2): through aberrant release of DAMPs, accumulation of metabolic intermediates, and transmission of retrograde stress signals, decompensated mitochondria trigger cascading responses along the dimensions of innate immunity, epigenetics, and organelle communication, progressively restricting the tissue's capacity for recovery.

**FIGURE 2:**
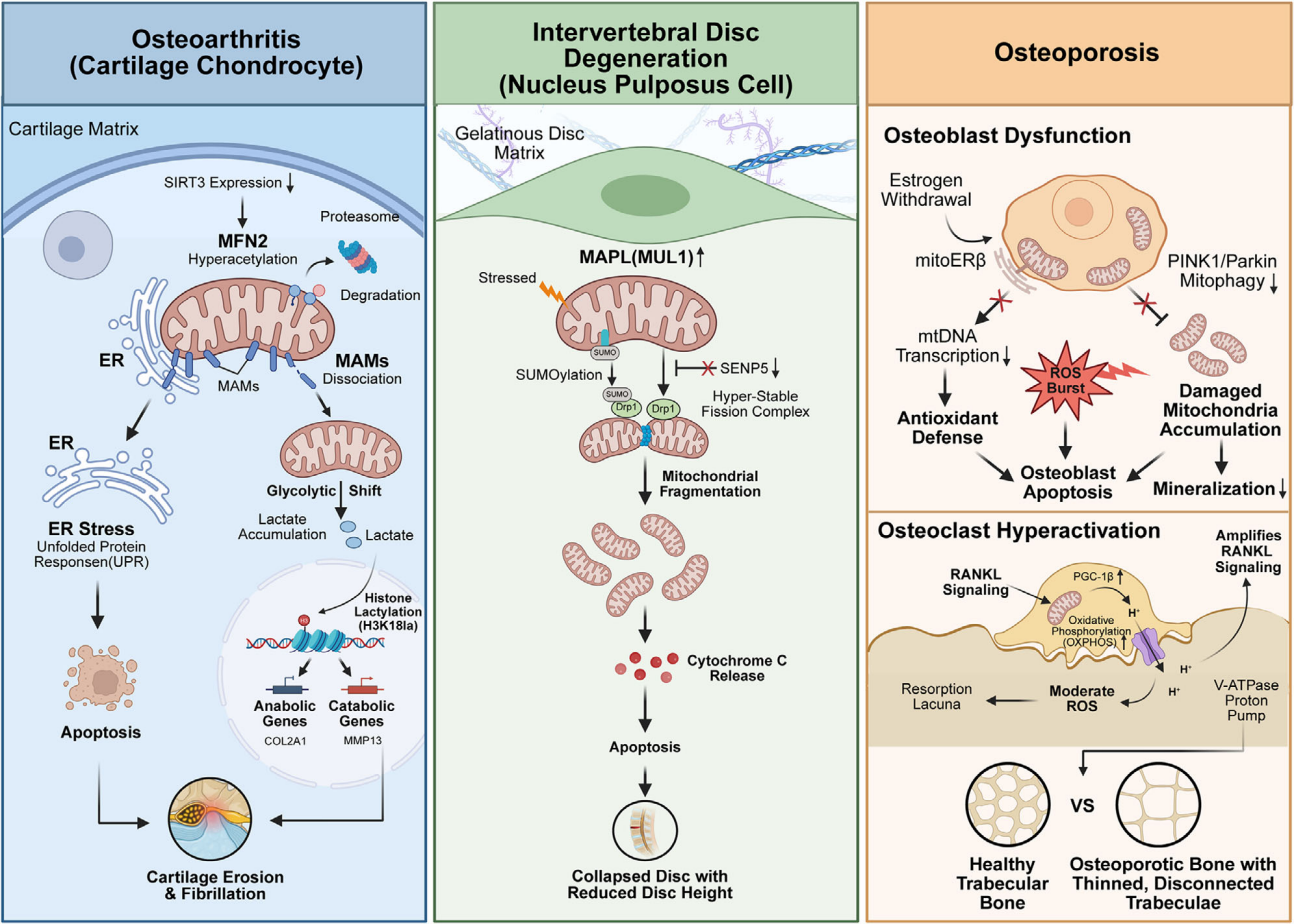
Cell‐type‐specific mechanisms of MQC dysregulation in different degenerative musculoskeletal diseases. The figure compares the MQC molecular defects of osteoarthritis, intervertebral disc degeneration, and osteoporosis in three parallel panels. **(Left)** Osteoarthritis (chondrocytes): SIRT3 downregulation leads to MFN2 hyperacetylation and ubiquitin‐proteasome degradation, causing disintegration of mitochondria‐ER contact sites (MAMs), which in turn triggers ER stress and the unfolded protein response (UPR); concurrently, mitochondrial dysfunction‐driven glycolytic shift causes lactate accumulation, driving histone lactylation (H3K18la), suppressing anabolic genes and activating catabolic genes, ultimately leading to cartilage erosion. **(Middle)** Intervertebral disc degeneration (nucleus pulposus cells): under stress, upregulation of the E3 ligase MAPL (MUL1) with downregulation of the deSUMOylase SENP5 leads to excessive SUMOylation of the fission protein Drp1, forming hyper‐stable fission complexes that cause extensive mitochondrial fragmentation, cytochrome c release, and apoptosis, ultimately resulting in disc collapse. **(Right)** Osteoporosis: involves coupling dysfunction of osteoblasts and osteoclasts. **(Upper)** In osteoblasts, estrogen withdrawal weakens mitochondrial estrogen receptor β (mitoERβ) signaling, and PINK1/Parkin‐mediated mitophagy is impaired, leading to accumulation of damaged mitochondria and ROS burst, triggering apoptosis and reduced mineralization. **(Lower)** In osteoclasts, RANKL signaling enhances PGC‐1β‐driven oxidative phosphorylation (OXPHOS) to fuel the V‐ATPase proton pump for acidifying the resorption lacuna; meanwhile, moderately elevated ROS further amplify RANKL signaling.

### 3.1 Osteoimmune Amplification: Innate Immune Sensitization and Cascade Activation Triggered by Mitochondrial DAMPs

According to the endosymbiotic theory, mitochondria are intracellular derivatives of ancient bacteria, and their core components (e.g., free mtDNA and cardiolipin), once exposed to the cytosol or extracellular environment, are strongly immunogenic to the host innate immune system [50]. In degenerative musculoskeletal diseases, the pathological mis‐triggering of this “self‐nonself” immune tolerance and recognition mechanism constitutes a central driver of local sterile inflammation [51].

#### 3.1.1 The MAPL‐Drp1 SUMOylation “Molecular Switch” in IVDD

In the hypoxic, nutrient‐deprived, and aberrantly loaded microenvironment characteristic of intervertebral disc degeneration, mitochondrial dynamics dysregulation is no longer merely enhanced fission but is redirected by specific post‐translational modifications. The aberrant upregulation of the OMM E3 ligase MAPL (MUL1) is a critical determinant of nucleus pulposus cell fate: mechanistic studies have confirmed that MAPL directly catalyzes SUMOylation of the fission executor Drp1 [52]. This modification recruits Drp1 to the OMM and stabilizes its oligomeric assembly by anchoring it at mitochondria‐ER contact sites (MAMs) [53]. These hyper‐stable fission complexes directly induce lethal mitochondrial fragmentation and cooperate with Bax/Bak to promote mitochondrial macropore formation, causing cytochrome c release and ultimately triggering nucleus pulposus cell apoptosis [54]. Under physiological conditions, the specific deSUMOylase SENP5 is responsible for timely shutdown of this switch; in degenerated discs, however, epigenetic silencing of SENP5 leads to persistent activation of the MAPL‐Drp1 axis, continuously driving irreversible cell death and extracellular matrix degradation [23].

#### 3.1.2 The cGAS‐STING‐IFN‐β Positive‐Feedback Inflammatory Circuit

When mitophagy is paralyzed and severely damaged mitochondria cannot be cleared in time, chronic oxidative stress induces pathological opening of the mitochondrial permeability transition pore (mPTP) or aberrant VDAC1 oligomerization, causing direct mtDNA leakage into the cytosol [55]. These ectopic double‐stranded DNA molecules are recognized by the cytosolic DNA sensor cyclic GMP‐AMP synthase (cGAS), which catalyzes synthesis of the second messenger cGAMP and strongly activates the ER‐resident stimulator of interferon genes (STING) [56]; activated STING recruits and phosphorylates downstream kinases TBK1 and interferon regulatory factor 3 (IRF3), driving transcription of type I interferons (especially IFN‐β) [57]. In the pathological microenvironments of OA and OP, elevated IFN‐β exerts dual adverse effects: through autocrine signaling it accelerates cellular senescence and induces widespread release of senescence‐associated secretory phenotype (SASP) factors, whereas through paracrine signaling it suppresses osteoblast differentiation and aberrantly activates osteoclasts [58]. Persistent STING pathway activation forms a vicious positive‐feedback loop: downstream inflammatory factors such as TNF‐α further damage mitochondrial function, causing more mtDNA leakage and thereby establishing a self‐sustaining osteoimmune amplification within the tissue [59].

#### 3.1.3 mtDNA‐Driven Macrophage Polarization Dysregulation

The injurious effects of leaked mtDNA are not confined to the originating cell; mtDNA acts as a key danger messenger that remodels the entire tissue immune microenvironment. Around synovitis and osteolytic foci, extracellular mtDNA is massively internalized by surrounding macrophages through phagocytosis or exosome‐mediated uptake, potently driving their polarization toward the destructive pro‐inflammatory M1 phenotype [60]. Mechanistically, overactivation of the cGAS‐STING pathway in macrophages triggers profound metabolic reprogramming: it markedly suppresses mitochondrial oxidative phosphorylation (OXPHOS), forcibly locking cellular metabolism into an inefficient glycolytic state, thereby blocking the transition toward the tissue‐reparative M2 phenotype [61]. This pathological “metabolic‐immune stalemate” constitutes the intrinsic logic underlying the persistence of chronic synovitis and the complete failure of injured tissue repair [62]. These observations suggest that clearing extracellular mtDNA from the microenvironment or blocking the STING signaling axis could break this stalemate and restore osteoimmune microenvironment homeostasis.

The evidence base for the above mechanisms is uneven: cGAS‐STING signaling is well established in innate immunity (established), whereas its activation and pathogenic role in OA and OP tissue currently rest on in vitro and animal‐model studies (emerging); the dual regulation of bone formation and resorption by IFN‐β and the macrophage metabolic‐immune stalemate derive mainly from animal models and cellular experiments (emerging); and the evidence for epigenetic silencing of SENP5 driving persistent MAPL‐Drp1 axis activation remains limited to single‐center studies. The relative contributions of the two pathways of mtDNA leakage (via mPTP opening or VDAC1 oligomerization) remain controversial, and the specificity of circulating mtDNA as an inflammatory trigger has been questioned. Future studies should provide direct evidence of in situ cGAS‐STING activation in human synovial or bone tissues, establish dose‐response relationships between IFN‐β levels and bone microenvironment damage, and evaluate the long‐term safety of STING‐targeted interventions in the osteoimmune microenvironment.

### 3.2 Metabolic‐Epigenetic Crosstalk: Nuclear Retrograde Signaling of Mitochondrial Dysfunction

Beyond directly mediating apoptosis and immune responses, mitochondria can transmit retrograde signals to the nucleus by releasing specific metabolic intermediates [63]; this process directly reshapes the chromatin epigenetic landscape, transcribing transient metabolic stress into long‐term cellular “pathological memory”.

#### 3.2.1 Glycolytic Reprogramming and the Epigenetic Effects of Metabolic Byproducts: UGDH and Histone Lactylation

In degenerative microenvironments such as OA, reduced mitochondrial function forces chondrocytes to undergo metabolic reprogramming and rely heavily on glycolysis, leading to massive intracellular accumulation of lactate [64]. Lactate is no longer regarded merely as a metabolic waste product but as a key donor for epigenetic modifications [65]. Elevated lactate concentrations drive lactylation (Kla) of specific lysine residues of UDP‐glucose dehydrogenase (UGDH): this modification directly inhibits UGDH activity in catalyzing synthesis of the hyaluronic acid precursor UDP‐glucuronic acid, impairing normal cartilage matrix synthesis; more importantly, lactylated UGDH undergoes aberrant nuclear translocation, destabilizing the nuclear STAT1 transcriptional complex and instead activating the MAP3K8‐MAPK signaling pathway, promoting widespread expression of inflammatory factors [66]. This intra‐articular cascade has so far been described in a small number of studies (cultured chondrocytes and human cartilage explants), with modest sample sizes, and awaits independent replication. Lactate, through its derivative lactyl‐CoA, is the donor for p300‐catalyzed lactylation of histone H3 at lysine 18 (H3K18la); this mark is enriched at the promoter regions of senescence‐ and fibrosis‐related genes [67], consolidating the degenerative fate of chondrocytes at the chromatin level. It remains unclear whether this axis operates in vivo as it does in cultured chondrocytes. The quantitative relationship between local lactate concentrations and UGDH lactylation in human cartilage is likewise unknown. Until such evidence is available, the axis is best regarded as a promising but unconfirmed branch of the metabolic‐epigenetic network.

#### 3.2.2 Collapse of Organelle Communication: The SIRT3‐USP47‐MFN2 Axis and ER Stress Cascade

Physical disintegration of mitochondria‐associated membranes (MAMs) is an early hallmark of degenerative pathology [68]. As described in Section 2.3.1, MFN2 is a key anchoring protein maintaining MAMs connections, and its downregulation causes dissociation of ER‐mitochondria contacts; this section further clarifies the upstream regulatory mechanism. In healthy skeletal cells, the mitochondrial deacetylase SIRT3 deacetylates and activates the deubiquitinase USP47, protecting MFN2 from excessive ubiquitin‐proteasome degradation [69]. Under the dual pressure of aging and chronic inflammation, however, SIRT3 expression is markedly downregulated, MFN2 remains hyperacetylated and is rapidly degraded, and MAMs physical connections are completely disrupted [70]. This collapse of organelle communication blocks normal Ca^2+^ transfer from ER to mitochondria, impairing TCA cycle energy efficiency, and causes massive accumulation of unfolded proteins in the ER lumen: persistent ER stress strongly activates the unfolded protein response (UPR), ultimately triggering apoptosis through the CHOP pathway [71]. These mechanisms together establish the SIRT3‐USP47‐MFN2 axis as a central regulator of organelle interaction homeostasis.

#### 3.2.3 Succinate Accumulation‐Mediated HIF‐1α Pseudohypoxia Signaling and Transcriptional Amplification Circuit

Dysfunction of mitochondrial respiratory chain complex II (succinate dehydrogenase, SDH) severely disrupts the tricarboxylic acid cycle, causing massive intracellular accumulation of the metabolic substrate succinate [72]. Leaked into the cytosol, succinate acts as a competitive inhibitor that directly blocks the catalytic activity of prolyl hydroxylases (PHDs), suppressing ubiquitination‐dependent degradation of hypoxia‐inducible factor (HIF‐1α) [73]; even in a fully oxygenated joint cavity microenvironment, cells remain in a pathological “pseudohypoxic” state. Aberrantly stabilized HIF‐1α translocates to the nucleus, binds promoter regions of genes such as IL‐1β, and drives potent inflammatory cascades in macrophages and synovial cells, inducing aberrant pannus formation and markedly aggravating structural destruction of the joint. The pathological actions of succinate are pleiotropic: beyond stabilizing HIF‐1α, it also competitively inhibits multiple histone demethylases of the KDM5 family; nuclear succinate accumulation can raise activating marks such as H3K4me3 and thereby amplify transcription of pro‐inflammatory genes such as IL‐6. The enzymatic basis of this inhibition was first defined in tumour models of FH/SDH deficiency [74] and has since been confirmed in cell‐free and cellular systems. By contrast, the intra‐articular circuit has not been demonstrated in situ in human cartilage or disc. It is currently extrapolated from macrophage and tumour studies. KDM5 expression and function have likewise not been examined systematically in human bone or cartilage, where the few available studies concern fracture healing rather than joint pathology [75]. Whether joint succinate accumulation raises H3K4me3 at pro‐inflammatory promoters remains to be tested. The quantitative relationship between local succinate concentrations and H3K4me3 occupancy in human tissue is likewise unknown.

### 3.3 Metabolic Memory and Mitochondrial Epigenetics: From Acute Stress to Chronic Fate

Degenerative musculoskeletal diseases run a protracted course, yet the metabolic stresses implicated in their onset are typically transient and episodic [76]. This discrepancy suggests that cells retain a record of past stress, and that this record, rather than the stress itself, sustains the pathological phenotype. This section addresses that possibility in three steps. We first define metabolic memory and assess the mitochondrial genome as its carrier. We then trace how metabolic intermediates reshape the epigenetic landscape and lock cells into a fixed state. We finally grade the available evidence and set out the validation route required to move the concept from hypothesis toward a testable clinical claim.

#### 3.3.1 Metabolic Memory: Concept and Mitochondrial Carrier

For the sake of subsequent discussion, this review defines this phenomenon uniformly as follows: metabolic memory is a state in which pathological cellular states persist even after the acute stress has resolved, triggered by MQC collapse and driven by epigenetic modifications induced by metabolic intermediates [77]. The definition contains three key elements: the trigger is MQC collapse, the driver is metabolic intermediate‐induced epigenetic modification, and the core consequence is persistence of the pathological state.

This memory is inscribed not only in nuclear chromatin but also directly encoded in the mitochondrial genome (mtDNA) itself. As the most direct memory carrier, mtDNA cytosine methylation (5mC) and hydroxymethylation (5hmC) levels are regulated by the mitochondrial one‐carbon metabolism network and TET enzyme homologs [78]; under persistent oxidative stress, regulatory regions such as the mtDNA D‐loop undergo aberrant hypermethylation, significantly suppressing their transcriptional activity [79], causing severe impairment of respiratory chain complex core subunit synthesis; even after the initial oxidative stress source is removed, mitochondrial energy output remains persistently impaired [80], forming a “vicious cycle of functional deficiency” that spans the stress event.

The existence and functional significance of mtDNA methylation remain contested, largely because detection is technically demanding in musculoskeletal tissue. Bisulfite sequencing is confounded by incomplete conversion and by the near‐identical mitochondrial sequences embedded in the nuclear genome (NUMTs). The generally low abundance of mitochondrial 5mC further limits the sequencing depth available after enrichment [81]. Antibody‐based enrichment such as MeDIP‐seq is sensitive to antibody specificity, and mitochondrial sequences can mislead global methylation measured in this way [82]. Mass spectrometry requires microgram amounts of purified mtDNA that small, cell‐heterogeneous biopsies cannot provide, and no cross‐laboratory standard exists [81]. Against this technical background, positive reports coexist with rigorous negative studies [83], and platform‐dependent artifacts may account for much of the discrepancy. We accordingly treat mtDNA methylation as a hypothesis‐driven extension of the framework rather than a core tenet. Its causal link to long‐term functional failure still awaits confirmation in human data.

#### 3.3.2 Metabolic Intermediates Reshape the Epigenetic Landscape

Mitochondria‐derived metabolic intermediates can also serve as direct “substrates” or “allosteric modulators” of epigenetic modifications, deeply “writing” aberrant metabolic states into nuclear chromatin [84]. Beyond lactate and succinate described above, the metabolic balance between α‐ketoglutarate (α‐KG) and 2‐hydroxyglutarate (2‐HG) constitutes another key regulatory axis: α‐KG is an indispensable cofactor for multiple nuclear histone demethylases (KDMs) and DNA demethylases (TETs) [85]; when the mitochondrial TCA cycle is impaired, α‐KG production becomes severely insufficient while its competitive inhibitor 2‐HG (which can be induced by specific mutations or metabolic stress) relatively accumulates [86]; this substrate‐inhibitor imbalance is expected to raise methylation of histones (e.g., H3K4me3, H3K9me3) and DNA [87]. This epigenetic “lock” state maintains high expression of pro‐inflammatory pathways such as NF‐κB and matrix‐degrading enzymes such as MMP13, while completely silencing the anabolic genes required for tissue repair [88].

Against this background, the chronicity of musculoskeletal disease may be understood in part as pathological epigenetic fixation [89]. This framework explains why anti‐inflammatory therapies in clinical practice often provide only transient symptom relief without reversing tissue degeneration. Targeting this core mechanism, emerging intervention strategies aim to break epigenetic fixation: for example, developing small‐molecule drugs targeting the mitochondria‐nucleus epigenetic axis (α‐KG supplementation or specific metabolic enzyme inhibitors) to restore normal epigenetic modification balance [90]; meanwhile, exploring epigenetic “erasers” to clear harmful metabolic memory marks such as H3K18la (see Section 3.2.1) at early disease stages, enabling cells to regain metabolic and differentiation plasticity [91]. If these strategies are validated in preclinical and clinical studies, intervening in “metabolic memory” may shift musculoskeletal disease treatment from “delaying progression” toward “pathological reversal”. This mechanism suggests the existence of a therapeutic window for resetting cell fate before “metabolic memory” becomes irreversibly fixed [92], providing a molecular biological rationale for early protective intervention at subclinical stages.

#### 3.3.3 Evidence Grading and Validation Route

The evidence base for the metabolic memory hypothesis is uneven. The regulation of KDM/TET enzymes by α‐KG/2‐HG is supported by mature enzymological and oncological work (established). Its pathological role within musculoskeletal joints, by contrast, rests on emerging research (emerging). Detection and functional studies of mtDNA methylation (5mC/5hmC) remain at an early stage and are controversial (controversial). Some studies question the reproducibility of the 5mC signal, pointing to nuclear DNA contamination and methodological artifacts [82, 83]. The causal chain from mtDNA methylation to long‐term functional failure has not been established in human data, and no clinical phenotypic biomarker of metabolic memory yet exists. Should mtDNA methylation signals ultimately prove to be technical artifacts or biologically negligible, the model would be narrowed rather than invalidated. Its nuclear and metabolic components would remain, since persistent metabolic imbalance, histone modification, and the self‐sustaining inflammatory circuit each rest on independent evidence.

These evidence gaps define a priority research agenda. The abundance and functional consequences of mtDNA methylation in bone and cartilage cells remain to be clarified. α‐KG supplementation and metabolic enzyme inhibitors require clinical evaluation in the joint field, and the delivery and specificity of epigenetic erasers are unresolved. Progress on all three will depend mainly on longitudinal human data rather than on further cross‐sectional observation. We propose a prospective, multicenter cohort. Surgical specimens of cartilage, disc, and bone would be paired with baseline and serial follow‐up over 12–24 months. Outcomes would cover imaging progression, symptom scores, and circulating and synovial‐fluid biomarkers. Paired sampling would relate local epigenetic marks to systemic signals, and standardized detection with explicit NUMT exclusion should be fixed before data collection begins. Biochemical readouts should be pre‐specified separately from clinical endpoints, because demonstrating biochemical memory does not by itself establish clinical relevance. This design is complementary to the OAI and MOST validation route described in Section 4.3.4.

### 3.4 Energy Competition and Sexual Dimorphism: The Specialized Microenvironment of Osteoporosis

In the pathological process of osteoporosis, the MQC network exhibits pronounced cell‐lineage specificity and heterogeneous metabolic demands [93]; concurrently, the postmenopausal decline in sex hormones reshapes the intracellular mitochondrial homeostasis network, contributing to a microenvironment with distinctive energy metabolism and sexual dimorphism in this disease [93, 94].

#### 3.4.1 The Energy/ROS Competition Between Osteoblasts and Osteoclasts

The physiological process of bone remodeling depends on the dynamic balance between osteoblasts (bone formation) and osteoclasts (bone resorption), which is a precisely intercellular “energy competition”. Osteoclasts, as typical high‐energy‐demanding cells, rely heavily on PGC‐1β‐driven mitochondrial biogenesis and efficient oxidative phosphorylation (OXPHOS) to continuously fuel the vacuolar H^+^‐ATPase (V‐ATPase) proton pump on the plasma membrane, executing potent bone matrix resorption [95, 96]. Under pathological conditions of osteoporosis, osteoclast MQC dysregulation often manifests as a moderate elevation of reactive oxygen species (ROS); this sublethal oxidative signal can act as a second messenger that amplifies RANKL‐mediated osteoclast differentiation and promotes bone resorption [97]. In stark contrast, osteoblasts are highly sensitive to the ROS microenvironment: as described in Section 2.2, defects in PINK1/Parkin‐mediated mitophagy lead to massive accumulation of damaged mitochondria and ROS burst, directly triggering osteoblast apoptosis and suppressing matrix mineralization capacity [34]. An ideal metabolic therapeutic strategy must therefore find a narrow therapeutic window that balances “suppressing osteoclast energy hyperactivity” against “restoring osteoblast mitochondrial health”.

#### 3.4.2 Dysfunction of Mitochondrial Estrogen Receptor Coupling

The pathogenesis of postmenopausal osteoporosis involves, beyond the loss of classical nuclear receptor signaling, a proposed contribution from uncoupling of the mitochondrial estrogen receptor (mitoER) axis [94]. Studies suggest that estrogen receptors (particularly ERβ) localize to the mitochondrial matrix and act on estrogen‐response‐element‐like sequences in mtDNA to regulate cytochrome c oxidase (COX) subunit expression and antioxidant enzymes such as manganese superoxide dismutase (MnSOD) [98]. This evidence derives largely from in vitro work, chiefly binding assays with recombinant human ERα/ERβ and MCF‐7 mitochondrial extracts, together with a few tissue studies in non‐skeletal organs [99]; estrogen deficiency also drives mitochondrial dysfunction in skeletal cells, as reported for endplate chondrocytes exposed to vertebral BMSC‐derived extracellular vesicles [98]. The abrupt withdrawal of estrogen at menopause is predicted to weaken this mitochondrial protective axis, which would expose osteoblasts to bioenergetic crisis and oxidative stress while releasing estrogen‐mediated restraint on osteoclast mitochondrial activity and favoring bone resorption. Direct evidence in human bone remains scarce. Conclusions about osteoblast mitochondrial regulation therefore rest on analogical inference from other tissues, and the functional role of mitoER binding to mtDNA has not been quantified in vivo. These caveats do not negate the translational interest of the axis. Restoring non‐genomic mitoER signaling remains an attractive and mechanistically rational intervention target for postmenopausal osteoporosis, provided that skeletal‐specific validation is prioritized before clinical extrapolation.

## 4 From Mechanisms to Clinical Translation: Precision Diagnosis and Molecular Stratification Based on Multi‐Omics Atlases

The core bottleneck in translating breakthroughs in fundamental MQC research into clinical value lies in developing high‐precision diagnostic tools capable of sensitively capturing early microscopic signs of organelle dysfunction. Conventional orthopedic imaging (X‐ray, MRI) depicts macroscopic tissue structure, and by the time structural changes become visible, the underlying cellular metabolic disturbance is already well established [100]. Accordingly, translational research increasingly integrates “liquid biopsy” [101] with high‐resolution “spatiotemporal multi‐omics” technologies [102] to construct molecular pathological maps of degenerative musculoskeletal diseases (Table 1), advancing clinical practice from lagging “anatomical diagnosis” toward early, precise “molecular subtyping” [103].

**TABLE 1:** Biomarkers for MQC‐related precision stratification and their clinical utility.

Biomarker category	Specific biomarker	Sample source	Reflected pathological process	Potential clinical utility
Nucleic acids	Circulating cell‐free mtDNA (ccf‐mtDNA) [104]	Plasma, synovial fluid, serum	Mitochondrial integrity collapse; cell necrosis/pyroptosis; DAMP‐mediated immune activation	1. Disease activity and prognosis assessment; 2. Immune activation status monitoring
Metabolites	Lactate [64]	Synovial fluid, tissue (spatial metabolomics)	Degree of glycolytic reprogramming; substrate for histone lactylation	Mapping “metabolic hotspots” co‐localized with matrix degradation zones
	Succinate [73]	Synovial fluid, tissue (spatial metabolomics)	Mitochondrial complex II dysfunction; pseudohypoxic signaling (HIF‐1α stabilization)	Identifying “inflammatory islands” co‐localized with synovial inflammation
	α‐Ketoglutarate/2‐hydroxyglutarate ratio [85]	Cells, tissue	TCA cycle function and global cellular methylation potential	Assessing epigenetic dysregulation: ratio imbalance indicates histone/DNA methylation abnormalities
Proteins and cytokines	cGAMP [59]	Synovial fluid, cell supernatant	Direct product of cGAS‐STING pathway activation	1. Core biomarker defining the “immuno‐inflammatory” subtype; 2. Pathway activity assessment
	Type I interferons (e.g., IFN‐β) [57]	Synovial fluid, blood	Downstream product of cGAS‐STING pathway; drives cellular senescence and immune dysregulation	1. Inflammation activity indicator; 2. Hub biomarker linking innate immunity and cellular senescence
	Core SASP factor panel (IL‐6, IL‐1β, MMP‐3, etc.) [105]	Blood, synovial fluid, tissue	Senescent cell burden and paracrine effects	1. Defining the “senescent cell accumulation” subtype; 2. Evaluating senolytic/anti‐aging strategy efficacy
Cell/functional indicators	Mitochondrial membrane potential (ΔΨm)	Tissue biopsy (ex vivo), fluorescence imaging	Core indicator of mitochondrial energy‐generating function	Cell‐level functional assessment: directly evaluating MQC‐targeted intervention efficacy
	Autophagic flux markers (LC3‐II/p62 ratio) [106]	Tissue, cells	Patency of autophagy (including mitophagy)	Assessing autophagy activation or blockade status

### 4.1 A New Dimension of Liquid Biopsy: Circulating Cell‐Free mtDNA as a “Molecular Sentinel” of Mitochondrial Crisis

When resident cells in the skeletal microenvironment undergo necrosis or pyroptosis following complete MQC network collapse, the mitochondrial genome, normally protected by double‐membrane structures, is released extracellularly and eventually enters the peripheral circulation, forming circulating cell‐free mtDNA (ccf‐mtDNA) [50]. Unlike non‐specific inflammatory markers such as C‐reactive protein (CRP) and erythrocyte sedimentation rate (ESR), ccf‐mtDNA is released when mitochondrial integrity is compromised and has been proposed as a candidate sentinel of early metabolic stress [107]; its specificity and its ability to report tissue‐level damage still require validation (Section 4.1.1). The mechanisms by which ccf‐mtDNA activates immunity as a DAMP are detailed in Section 3.1; this section focuses on its diagnostic value as a biomarker.

#### 4.1.1 Pathological Correlations, Prognostic Value, and Detection Parameters

Recent large‐scale clinical cohort studies have shown that ccf‐mtDNA concentrations in the synovial fluid and plasma of OA patients correlate significantly with radiographic joint degeneration grading and tightly with the severity of local synovitis and the rate of cartilage matrix loss [108]. In osteoporosis, elevated ccf‐mtDNA has been proposed as a systemic readout of osteoblast mitochondrial dysfunction [109]. Preliminary evidence suggests that it may add predictive information on fragility fracture risk in postmenopausal women beyond bone mineral density (BMD), although prospective confirmation is required. As a detection indicator, quantification and interpretation of ccf‐mtDNA require attention to three technical dimensions: (1) fragment length: ccf‐mtDNA fragments are generally shorter than nuclear cfDNA and are influenced by tissue origin and degradation status; (2) methylation status: ccf‐mtDNA carries CpG motifs that differ in methylation from nuclear cfDNA, and methylation‐based inference of tissue of origin, which is established for nuclear cfDNA, remains to be validated for ccf‐mtDNA; (3) mt/nDNA ratio: a quantitative indicator reflecting mitochondrial stress intensity, although amplification interference from mitochondrial pseudogenes in the nuclear genome (NUMTs) must be excluded. Standardization of these parameters is a prerequisite for ccf‐mtDNA to enter routine clinical testing, and a unified consensus is currently lacking.

#### 4.1.2 Immune Activation Biomarkers

Because ccf‐mtDNA is enriched in hypomethylated CpG motifs, it can act as a highly bioactive DAMP in the circulation, persistently activating TLR9 and cGAS‐STING innate immune pathways (mechanisms detailed in Section 3.1.2). Serial measurement of ccf‐mtDNA may allow mitochondrial damage and innate immune activation to be tracked over time, and could help identify patients with rapidly progressing disease who are most likely to benefit from anti‐inflammatory or mitochondrial‐protective therapy; whether it carries predictive value remains to be established (Section 4.3.4).

### 4.2 Decoding Heterogeneity: Single‐Cell and Spatial Multi‐Omics Reshape the Pathological Microenvironment Map

Degenerative musculoskeletal tissues exhibit extreme spatiotemporal complexity in cellular composition and dynamic metabolic networks [110]. Conventional bulk RNA‐seq masks the features of key rare subpopulations driving disease progression and blurs the spatial topology of organelle interactions [89]. Single‐cell and spatial multi‐omics technologies are now reconstructing the pathological microenvironment map at much higher resolution.

#### 4.2.1 scRNA‐Seq‐Defined “Mitochondrial Stress” Subpopulations

In‐depth scRNA‐seq analysis has identified a highly specific subpopulation of “mitochondrial dysfunction‐associated regulatory chondrocytes” (Mito‐regulatory Chondrocytes) in OA cartilage and degenerated disc tissue [111]. This subpopulation exhibits a clear molecular boundary with classical senescent cells (SnCs): it aberrantly overexpresses glycolytic rate‐limiting enzymes (e.g., HK2, LDHA) and the mitochondrial fission executor gene DNM1L, while systematically silencing PGC‐1α and its downstream OXPHOS gene clusters [112]. Although this subpopulation accounts for only a small fraction of tissue cells, it is a major source of pro‐inflammatory SASP factors (e.g., IL‐1β, MMP13) in the microenvironment and may act as a pathological epicenter driving tissue degeneration [113].

#### 4.2.2 Spatial Metabolomics Visualizes Metabolic Gradients

Spatial metabolomics technologies generate high‐resolution metabolite maps in situ on tissue sections, allowing metabolic features to be aligned directly with tissue pathology [114]. Investigators have delineated “lactate hotspots” in the deep layers of OA cartilage that overlap extensively with matrix degradation zones, and precisely localized high‐concentration “succinate islands” in severely inflamed synovium [66]. This integrative analysis shows that metabolite‐enriched regions co‐localize with histone lactylation (H3K18la) and HIF‐1α signaling activity [115], a pattern consistent with, but not proof of, a role for mitochondrial metabolic reprogramming in reshaping the epigenetic state of neighboring cells by altering local microenvironment chemistry [65].

### 4.3 From Biomarkers to Precision Stratification: Constructing an MQC‐Driven Patient Subtyping Framework

Multi‐omics atlases have revealed the extensive molecular heterogeneity underlying degenerative musculoskeletal diseases. This heterogeneity limits the reach of traditional “one‐size‐fits‐all” intervention strategies [116]. Precision medicine now aims to translate these biomarkers into an actionable patient molecular stratification framework, ultimately enabling individualized therapy [117]. Using OA as an example, this review proposes a precision subtyping prototype centered on MQC network status, initially dividing the disease into the following three targetable subtypes.

#### 4.3.1 Mitochondrial Bioenergetic Failure Subtype

The core molecular portrait of this subtype is a compensatory moderate elevation of ccf‐mtDNA levels in synovial fluid or peripheral blood, accompanied by systematic transcriptional downregulation of PGC‐1α and its downstream OXPHOS gene clusters [103]; musculoskeletal ultrasound or MRI may capture concomitant early muscle atrophy, suggesting involvement of skeletal muscle mitochondrial function [108]. The core pathological driver is a generalized defect in cellular energy production, directly causing arrest of chondrocyte anabolic metabolism [93]. Priority intervention strategies should focus on energy remodeling, including AMPK activators (e.g., metformin) [119], PGC‐1α agonists, NAD^+^ precursors (e.g., NMN/NR) [120], and mRNA delivery therapies that promote mitochondrial biogenesis in a targeted manner. The association of this subtype with energy depletion markers is supported by initial cohort and omics evidence (emerging); its comorbidity relationship with sarcopenia warrants prospective validation.

#### 4.3.2 Immuno‐Inflammatory Activation Subtype

The hallmark feature of this subtype is markedly elevated concentrations of cGAMP (a cGAS catalytic product), IFN‐β, and specific chemokines in synovial fluid [59], with patients often presenting peak ccf‐mtDNA levels and strong cGAS‐STING pathway activation on tissue biopsy. The pathological root lies in massive mtDNA leakage triggering a persistent innate immune inflammatory cascade, driving sterile inflammation and accelerated cellular senescence [121]. Intervention strategies should prioritize immune‐metabolic blockade, including STING small‐molecule inhibitors [122], mitochondrial‐targeted membrane stabilizers (e.g., SS‐31) to reduce danger signal release [123] (clinical evidence detailed in Section 5.2.1), and therapies that clear extracellular mtDNA (e.g., recombinant DNase I analogs) [124]. The cGAMP/IFN‐β biomarker combination of this subtype is supported by initial biomarker studies (emerging); evidence for in situ activation of the STING pathway in human joint and bone tissues remains to be accumulated.

#### 4.3.3 Senescent Cell Accumulation Subtype

Histologically, this subtype manifests as strongly positive enrichment of senescence‐associated β‐galactosidase (SA‐β‐Gal) activity; blood or synovial fluid analysis reveals high expression of a characteristic SASP factor profile (e.g., IL‐6, MMP‐3, PAI‐1), while elevated LC3‐II/p62 ratios are consistent with impaired autophagic degradation, although flux cannot be inferred from steady‐state markers alone [113]. The core pathological mechanism is local accumulation of dysfunctional senescent cells that continuously “poison” the surrounding healthy microenvironment through paracrine SASP [125]. Priority recommendations include senolytic drug combinations (targeted elimination of anti‐apoptotic cells) [126], senomorphics (e.g., the p300/CBP inhibitor A485 suppressing SASP transcription) [127], and potentially combined lysosomal acidification activators to reopen the obstructed autophagic clearance network. The SA‐β‐Gal and SASP evidence for this subtype is relatively robust (between established and emerging); application standards for LC3‐II/p62‐based autophagic flux assessment in human joint tissue have not yet been established.

#### 4.3.4 Evidence Base Distinction, Validation Routes, and Trial Design

The evidence bases of the three subtypes are asymmetric and must be honestly distinguished: the biomarker combinations of the bioenergetic failure and senescent cell accumulation subtypes are supported by relatively more omics and clinical studies (emerging‐established); the cGAMP/IFN‐β combination of the immuno‐inflammatory subtype is newer with limited sample size (emerging); and associations of all three subtypes with clinical outcomes (imaging progression, symptoms, surgery rates) lack prospective validation. Validation routes (the following are recommended study designs, not existing data): using stored specimens from public cohorts such as the Osteoarthritis Initiative (OAI) and the Multicenter Osteoarthritis Study (MOST), unsupervised clustering or machine learning methods should be applied to test the stability of the three‐subtype classification and to correlate with imaging progression, symptom scores, and prognosis; if clustering results do not support the established boundaries, the subtyping should be revised accordingly.

Trial design example: for the immuno‐inflammatory activation subtype, a biomarker‐enriched trial of a STING inhibitor combined with SS‐31 could be designed, with enrollment criteria of synovial fluid cGAMP/IFN‐β above a set threshold and primary endpoints covering structural (cartilage volume by MRI), molecular (cGAMP decline), and functional (Western Ontario and McMaster Universities Osteoarthritis Index, WOMAC) scores, with 12–24 months of follow‐up. The above validation routes are recommended study designs intended to provide an empirical basis for the three‐subtype classification; they do not represent existing data (Table 2).

**TABLE 2:** MQC subtype‐biomarker combination table.

Subtype	Candidate biomarker combination	Preferred sample type	Current evidence status
Bioenergetic failure	ccf‐mtDNA (moderately elevated), PGC‐1α/OXPHOS transcriptional downregulation [104]	Plasma, synovial fluid	emerging (initial cohort and omics support)
Immuno‐inflammatory activation	cGAMP, IFN‐β, chemokines, ccf‐mtDNA (peak) [59]	Synovial fluid	emerging (initial biomarker studies)
Senescent cell accumulation	SA‐β‐Gal, SASP profile (IL‐6/MMP‐3/PAI‐1), LC3‐II/p62 [113]	Synovial fluid, tissue biopsy	emerging‐established

These validation routes address subtype stability within a single disease. A further question concerns their cross‐disease generalizability. The three‐subtype classification was developed principally from knee OA, and whether the same boundaries, biomarker combinations, and therapeutic matches hold in IVDD and OP is currently unknown and cannot be assumed. Cross‐disease validation is therefore a prerequisite for extending the framework beyond OA; until it is available, the subtyping and treatment‐matching proposals advanced here should be read as disease‐specific. A subtype that stratifies patients within one disease but not across the three conditions would still be clinically useful. It would not, however, support the unified MQC‐centered disease concept proposed here. Direct comparisons of subtype stability, biomarker performance, and outcome associations across OA, IVDD, and OP cohorts should accompany any single‐disease validation, ideally within a single multi‐center protocol using identical analytical pipelines. Until such data exist, the subtyping framework is best regarded as a within‐OA hypothesis whose cross‐disease generalizability remains unproven.

The MQC subtyping framework aims to connect the basic mechanistic map with complex clinical decision‐making: it provides a theoretical benchmark for biomarker‐enriched clinical trial design and lays the foundation for maximizing targeted therapeutic efficacy, advancing orthopedic degenerative diseases toward individualized precision medicine. This framework remains a conceptual prototype, and its clinical value depends on the outcomes of the validation routes described above.

## 5 From Concept to Clinic: Therapeutic Strategies Targeting Mitochondrial Quality Control and the Translational Landscape

Clinical treatment strategies for degenerative orthopedic diseases are undergoing a fundamental transformation: from traditional passive defense based on “symptomatic anti‐inflammation” toward active intervention centered on “organelle functional remodeling” [128]. Targeted therapies now aim to precisely regulate key nodes of the MQC network, with intervention dimensions spanning mitochondrial ultrastructural stabilization, targeted clearance of pathogenic protein products, repair of damaged mtDNA, and even direct transplantation of healthy organelles [129]. Translating these preclinical concepts into safe and effective therapies, however, requires bridging the “translational gap” between fundamental mechanistic elucidation and real clinical practice [130]. This section reviews the molecular pharmacological basis and clinical translation progress of these intervention strategies and analyzes the core technical obstacles to their widespread clinical application.

### 5.1 The Spectrum of Precision Intervention Strategies

Building on the dissection of key nodes within the MQC network, a multi‐tiered intervention spectrum has now emerged, ranging from micro‐level molecular regulation to macro‐level organelle replacement [128]. The fundamental goal is to restore the metabolic homeostasis of skeletal cells at the most basic level, rather than passively suppressing downstream pathological symptoms.

#### 5.1.1 Precision Pharmacology: Cardiolipin‐Targeted Stabilization and Post‐Translational Modification Regulation

Traditional antioxidants such as vitamin C/E, lacking mitochondrial targeting and unable to reach effective concentrations within target organelles, have repeatedly failed in clinical translation, prompting precision pharmacological strategies centered on “structural stabilization” and “signal reprogramming”. Mitochondrial structural stabilizers: represented by the aromatic cationic tetrapeptide elamipretide (SS‐31), which specifically penetrates the mitochondrial double membrane and precisely anchors to cardiolipin enriched in the inner membrane [131]. In OA and ischemic osteonecrosis models, SS‐31 acts not merely as a ROS scavenger but, more importantly, as a “molecular scaffold” that stabilizes the cardiolipin‐cytochrome c complex, preventing aberrant activation of cytochrome c peroxidase activity and thereby maintaining the integrity of electron transport chain (ETC) supercomplexes and cristae structure [123]. This structural protection directly blocks mtDNA leakage into the cytosol and suppresses downstream cGAS‐STING immune pathway activation at its source [132], offering a pharmacological paradigm for breaking the “mitochondria‐inflammation” vicious cycle (clinical evidence detailed in Section 5.2.1). Post‐translational modification modulators: given the driving roles of lactylation, SUMOylation, and other modifications in pathological progression, small‐molecule drugs targeting pathological PTMs hold considerable potential [65]. The highly selective p300/CBP catalytic core inhibitor A485 effectively blocks aberrant H3K18la and non‐histone lactylation in OA chondrocytes, relieving transcriptional repression of anabolic genes and attenuating matrix degradation [133]. In therapeutic exploration for IVDD, given the off‐target risk of the broad‐spectrum Drp1 GTPase inhibitor Mdivi‐1 (which has been confirmed as a reversible inhibitor of mitochondrial complex I, with anti‐fission effects potentially independent of Drp1 [134]), research has pivoted toward more selective fission regulators. Allosteric inhibitors targeting specific Drp1 SUMOylation sites (e.g., the MAPL‐catalyzed site) are under active development, aiming to precisely intervene in the over‐activated fission “molecular switch” and rescue nucleus pulposus cells from apoptosis.

#### 5.1.2 Protein Degradation Strategies: PROTAC‐Mediated Clearance of Pathogenic Proteins

Proteolysis‐targeting chimeras (PROTACs) achieve complete degradation of pathogenic proteins by hijacking the endogenous ubiquitin‐proteasome system, overcoming the pharmacological limitations of traditional occupancy‐driven inhibitors [135]: one end of the molecule specifically binds the target protein while the other end recruits a specific E3 ubiquitin ligase (e.g., VHL or Cereblon), inducing polyubiquitination and degradation of the target [136]. In degenerative musculoskeletal diseases, aberrant overexpression of the OMM E3 ligase MAPL is the core driver of Drp1 hyperactivation and extreme mitochondrial fragmentation; designing MAPL‐targeting PROTACs holds promise for specifically knocking down its protein level and restoring mitochondrial dynamics balance [137]; for hyperactive Drp1 in osteoclasts, developing mitochondrial‐targeted mito‐PROTACs can precisely suppress pathological bone resorption while minimizing interference with the normal physiological functions of cytosolic Drp1 (such as peroxisome division) [138]. The clinical translation of PROTACs is constrained by significant pharmacokinetic, toxicological, and delivery challenges (see Section 5.3), and they currently remain at the early compound validation stage.

#### 5.1.3 Genomic and Nucleic Acid Medicine: Mutation Repair and Transient Reprogramming

For skeletal degenerative pathologies driven by inherited or somatically accumulated mtDNA mutations, the traditional CRISPR‐Cas9 system is limited by the difficulty of mitochondrial matrix delivery and its propensity to induce mtDNA double‐strand break degradation. Emerging nucleic acid medicine approaches are beginning to address this barrier. Mitochondrial base editing: the DddA‐derived cytosine base editor (DdCBE) has achieved the first precise single‐base conversion of C•G to T•A within the mitochondrial genome without double‐strand DNA cleavage [139], holding potential value for treating orthopedic complications of maternally inherited mitochondrial diseases (such as MELAS syndrome): by precisely correcting pathogenic mutations in respiratory chain subunits (e.g., ND1, ND4) or tRNAs, it may rebuild the bioenergetic supply network of osteoblasts from the genetic root [140]. mRNA‐LNP delivery systems: drawing on the clinical success of nucleic acid vaccines, modified mRNAs encoding mitochondrial homeostasis proteins (PGC‐1α, TFAM, SIRT3) or master transcription factors such as SOX9 can be encapsulated in lipid nanoparticles (LNPs) and delivered locally via intra‐articular injection [141]; this “transient gene therapy” that does not integrate into the host genome can substantially enhance mitochondrial biogenesis, antioxidant defense, and matrix synthesis of target cells during critical tissue repair windows [142]. DdCBE has so far been applied mainly in liver and muscle models; its application in bone cells remains preclinical, with safety, efficiency, and heterogeneity unresolved. Repeated dosing and immunogenicity of mRNA‐LNPs also await evaluation.

#### 5.1.4 Organelle Medicine: Engineered Mitochondrial Transplantation

When the intracellular mitochondrial pool suffers widespread irreversible damage, the most fundamental intervention is direct introduction of healthy mitochondria. Enhancing endogenous “mitochondrial donation”: in the skeletal microenvironment, mesenchymal stem cells (MSCs) can transfer functional mitochondria to damaged osteoblasts or chondrocytes through tunneling nanotubes (TNTs) or microvesicle release [143]; genetic engineering of MSCs to overexpress the motor protein Miro1 can significantly improve this endogenous donation efficiency and rescue the severe energy crisis of recipient cells [144]. Engineered artificial mitochondria: to overcome the physical limitation that naked mitochondria are easily inactivated in the extracellular environment, researchers have developed the “Fmito@ACs” technology, in which highly active mitochondria from fetal BMSCs are encapsulated in a highly biocompatible artificial cell membrane system [145]: the engineered organelles can evade immune recognition and clearance, and through surface modification with bone‐targeting peptides (tetracycline analogs or octa‐aspartate peptides) integrate into the recipient mitochondrial network, directly supplementing ATP production and reversing redox imbalance [146]. In preclinical models, this strategy extends regenerative medicine from cell transplantation toward subcellular component transplantation. Immunogenicity, long‐term safety, and scaled production remain the main obstacles to clinical translation.

#### 5.1.5 Smart Biomaterials and Biophysical Signal Modulation

To achieve efficient and controllable delivery of targeted drugs and non‐invasive MQC regulation within the extremely complex pathological microenvironment of skeletal tissue, the interdisciplinary integration of materials science and biophysics has provided new intervention tools. Microenvironment‐responsive delivery systems: smart hydrogels crosslinked by ROS‐sensitive chemical bonds (e.g., thioketal bonds, arylboronic ester bonds) or MMP‐cleavable substrates remain highly inert in healthy microenvironments and undergo specific degradation or swelling in response to locally elevated ROS or protease levels upon entering inflammatory lesions [147]. This enables “on‐demand release” of loaded mitochondrial protectants (such as MitoQ; clinical evidence detailed in Section 5.2.4) or gene‐silencing drugs, maximizing localized high‐concentration therapy while minimizing systemic side effects [148]. Biophysical signal modulation: physical interventions such as low‐intensity pulsed ultrasound (LIPUS) and pulsed electromagnetic fields (PEMF) have been reported to modulate mitochondrial metabolism as “drug‐free” stimuli: their mechanical or electromagnetic parameters trigger calcium influx through mechanosensitive ion channels (especially Piezo1) [149], activating the AMPK‐PGC‐1α axis [150], and promoting mitochondrial biogenesis in cells and animal models [151]. Whether they promote fracture healing or reverse cartilage degeneration in patients remains to be tested.

### 5.2 Clinical Translation Progress: Evidence Levels and Lessons Learned

The intervention strategies described above are at a critical translational juncture, moving from preclinical concept validation toward real clinical application [100]. This section systematically evaluates the clinical evidence levels of different intervention strategies (Table 3), analyzes in depth the key drivers of their success and the core lessons of clinical trial setbacks, and thereby outlines a rational blueprint for the future evolution of skeletal microenvironment‐targeted therapy.

**TABLE 3:** Evidence levels of therapeutic strategies.

Strategy	Mechanism of action	Disease indications	Evidence level	Model systems	Translation barriers	Clinical readiness
Metformin	AMPK activation upregulating PGC‐1α, promoting mitochondrial biogenesis [119]	Knee OA (RCT); OP (preclinical)	In clinical use (positive JAMA 2025 RCT)	Human RCT + mechanistic studies	Benefit magnitude, dose optimization	Under clinical validation
NAD^+^ precursors (NMN/NR)	NAD^+^ replenishment activating sirtuins, promoting mitochondrial protein deacetylation [120]	Sarcopenia, secondary OP	Early clinical (muscle mitochondrial function in healthy older women)	Human trials + animal models	Long‐term safety, tissue specificity	Early clinical
SS‐31 (Elamipretide)	Cardiolipin stabilization maintaining ETC integrity, blocking mtDNA leakage [152]	OA, ischemic osteonecrosis	Approved (Barth syndrome 2025); orthopedic: preclinical	Human (Barth) + joint animal models	Lack of large‐scale joint RCTs, delivery	Partial clinical
Senolytics (UBX0101)	Inducing senescent cell apoptosis [153]	Knee OA	Phase II failed (primary endpoint not met)	Human phase II + animal models	Metabolic quiescence conferring apoptosis insensitivity, dosing frequency	Failed/strategic pivot
Senomorphics (A485, etc.)	Suppressing SASP transcription (p300/CBP) [133]	OA	Preclinical	Cells + animals	Selectivity, delivery	Conceptual stage
PROTAC (incl. mito‐PROTAC)	Targeted protein degradation (MAPL, Drp1) [135]	IVDD, OP	Preclinical (early compound validation)	Cells + animals	Macromolecular delivery (>700 Da), pharmacokinetics/toxicology	Conceptual stage
Mitochondrial base editing (DdCBE)	mtDNA single‐base correction (C•G→T•A) [139]	MELAS‐related bone disease	Preclinical (mainly liver/muscle models)	In vitro + animals (non‐bone)	Efficiency, safety, heterogeneity, intra‐bone delivery	Conceptual stage
mRNA‐LNP	Transient expression of mitochondrial homeostasis proteins (PGC‐1α/TFAM/SIRT3) [141]	OA	Preclinical	Animal models	Repeated dosing, immunogenicity, delivery	Preclinical
Engineered mitochondrial transplantation	Direct supplementation of healthy mitochondria [146]	Bone defects	Preclinical	Animal models	Immunogenicity, long‐term safety, scaling	Conceptual stage
Smart hydrogels/biophysical	Microenvironment‐responsive delivery (MitoQ, etc.) or Piezo1‐AMPK regulation [123]	OA, fracture	Preclinical; MitoQ in multiple phase II trials (non‐orthopedic)	Animals + partial human	Lack of orthopedic evidence	Preclinical
Digital twin/AI	Virtual screening, enrichment trial design, dynamic treatment path optimization [154]	Pan‐disease assistance	Conceptual	Computational models	Data quality, external validation	Conceptual stage

#### 5.2.1 Elamipretide (SS‐31): From Cardiolipin Stabilization to Orthopedic Tissue Repair

Leveraging its well‐defined pharmacological mechanism of specifically binding and stabilizing cardiolipin and protecting ETC integrity, the mitochondrial‐targeted peptide SS‐31 has successfully bridged the gap from bench to bedside [152]. In 2025, the US FDA granted accelerated approval based on its significant efficacy on intermediate clinical endpoints such as improved knee extensor strength in patients with Barth syndrome (a severe cardiolipin metabolism disorder) [186]. In the field of orthopedic degenerative diseases, preclinical pharmacokinetic studies have confirmed that SS‐31 can effectively penetrate the dense avascular cartilage matrix, precisely localize to the inner mitochondrial membrane of chondrocytes, and reverse cartilage degeneration by inhibiting mtDNA leakage and blocking downstream cGAS‐STING pathway activation [155]. This representative case establishes the clinical feasibility of the core strategy of “targetedly stabilizing mitochondrial membrane structure and blocking downstream sterile inflammation”. The evidence for SS‐31 in the joint field still derives mainly from cardiac and genetic disease data plus joint preclinical studies, lacking direct large‐scale joint RCTs; the current core translational challenge is how to convert this transient subcellular protective effect into substantial, long‐term clinical symptomatic improvement in musculoskeletal patients, for example through intra‐articular sustained‐release microspheres to optimize delivery [156].

#### 5.2.2 Metformin: Beyond Metabolic Modulation: A Repurposed Drug

Metformin, a classic first‐line drug for type 2 diabetes, is gaining attention for its potential in bone metabolism regulation and microenvironment remodeling [157]. Its core mechanism involves specific activation of the AMPK signaling pathway, cascading upregulation of PGC‐1α expression, potently driving mitochondrial biogenesis and comprehensively optimizing ETC respiratory efficiency [158]. A randomized, double‐blind, placebo‐controlled trial published in JAMA in 2025 reported that oral metformin reduced knee pain in overweight or obese patients with symptomatic knee OA relative to placebo, an effect that remained below the 15‐mm threshold for clinical importance and requires confirmation in a larger trial [159]. The mechanism of this symptomatic benefit is uncertain; it has been attributed to improvement of systemic glucose and lipid metabolism and to remodeling of the local skeletal microenvironment, and was reported to be independent of weight loss. Mechanistic studies have confirmed that, through mitochondrial retrograde signaling, metformin induces osteoblast differentiation and mineralization while suppressing the energy metabolism hyperactivity of osteoclasts and reshaping the bone marrow immune microenvironment by promoting pro‐reparative M2 macrophage polarization [160]. The highly anticipated “Targeting Aging with Metformin” (TAME) large‐scale clinical trial is expected to provide more definitive evidence [161], validating whether systematically modulating the mitochondrial‐energy sensing network can fundamentally delay the broad aging process including musculoskeletal degeneration.

#### 5.2.3 The Lessons and Strategic Pivot of Senolytics: From Simple Clearance to Metabolic Reprogramming

Senolytic therapies, designed to selectively induce apoptosis of senescent cells, were once highly anticipated, but the small‐molecule MDM2 inhibitor UBX0101 failed to show efficacy superior to placebo on pain improvement endpoints in a phase II clinical trial for knee OA, marking a major setback for this direction [162]. Initial failure attribution analyses focused mostly on tissue heterogeneity, dosing frequency, and placebo effects, but these explanations lack MQC specificity. Deeper mechanistic reflection points to a key paradox: senescent cells are in a metabolically quiescent state, with significantly reduced mitochondrial oxidative phosphorylation activity, and both the bioenergetic supply required for apoptosis induction and the sensitivity of mitochondrial outer membrane permeabilization are correspondingly decreased. This has given rise to the “sequential therapy” hypothesis: first “awaken” senescent cell metabolism and restore mitochondrial function with NAD^+^ precursors or mitochondrial‐targeted antioxidants, then sequentially use senolytics to increase apoptosis induction sensitivity [153]. This direction is driving a strategic pivot of the field: from “Senolytics” that induce apoptosis toward “Senomorphics” that improve the compensatory functions of senescent cells (suppressing the transcriptional source of SASP), the latter potentially offering a wider safety window and greater long‐term feasibility in maintaining tissue homeostasis.

#### 5.2.4 NAD^+^ Precursors (NMN/NR) and Redox Modulators

With natural aging, the drastic depletion of intracellular NAD^+^ reserves leads to significantly reduced activity of the deacetylase (sirtuin) family, in turn causing widespread aberrant acetylation of mitochondrial proteins and comprehensive imbalance of the MQC network [120]. As direct metabolic precursors of NAD^+^, nicotinamide mononucleotide (NMN) and nicotinamide riboside (NR) have entered early‐phase human studies and preclinical models of musculoskeletal degeneration. In human skeletal muscle, five months of NR supplementation was associated with reduced epigenetic age acceleration, which correlated with mitochondrial content [187]; in a chemotherapy‐induced bone loss model, NMN largely prevented bone loss, an effect attributed to preservation of BMSC mitochondrial homeostasis and osteogenic potential [164]. Whether NAD^+^ precursors modify bone or joint degeneration in patients remains to be established. Mitochondrial‐targeted antioxidants represented by MitoQ, by virtue of the lipophilic triphenylphosphonium (TPP^+^) cation, precisely penetrate and accumulate in the inner mitochondrial membrane to scavenge superoxide anions in situ [165]; this drug is undergoing multiple clinical trials for endothelial dysfunction and chronic inflammatory diseases, and its potential to reverse OA synovitis and oxidative cartilage damage warrants further study.

#### 5.2.5 Sequential and Combination Therapeutic Strategies

Given the profound complexity of the pathological network of degenerative musculoskeletal diseases and the spatiotemporal dynamics of MQC regulatory mechanisms, expecting a single‐target intervention to achieve durable, disease‐modifying control is no longer sufficient; future clinical treatment paradigms must closely track the dynamic evolution of the disease cycle and adopt highly intelligent sequential and combination strategies [166]. Sequential therapeutic logic: the core principle is to flexibly shift the therapeutic focus according to the “principal contradiction” at different stages of disease progression: during acute inflammatory flare‐ups or rapid bone destruction phases, cGAS‐STING‐targeted immune blockers or nano anti‐inflammatory agents should be prioritized to rapidly suppress the inflammatory flare [60]; after local inflammation is effectively controlled, the golden window for repair and regeneration opens, and the therapeutic focus must fully shift toward “promoting metabolic regeneration”, prioritizing mitochondrial biogenesis mRNA delivery therapies, engineered mitochondrial transplantation, or SIRT3 activators. Combination therapeutic strategies: these aim to target multiple pathological crosstalk nodes simultaneously or sequentially to produce potent synergistic effects, for example combining PROTACs that clear pathogenic proteins with small‐molecule activators of protective pathways such as AMPK agonists, or sequentially applying senolytic therapies that clear pathological obstacles with mitochondrial modulators that provide a healthy metabolic niche for nascent stem cells [135]. Although combination therapy faces severe challenges of drug interactions, dose titration optimization, and the predictive accuracy of animal models, it is likely to be required if durable disease modification is to be achieved in degenerative musculoskeletal diseases.

### 5.3 Translational Core Challenges: Biological Barriers and Microenvironmental Heterogeneity

Clinical translation of MQC‐targeted therapy faces two core challenges: the biological delivery barriers posed by the specialized anatomical and physiological structures of the musculoskeletal system [167], and the molecular and cellular heterogeneity of the diseases themselves [100]. The former limits effective drug accumulation at target tissues, while the latter determines that a single strategy cannot cover the entire patient population; together they constitute the realistic bottleneck for MQC‐targeted therapy to move from concept to clinic.

Deep target tissues such as articular cartilage and the nucleus pulposus of intervertebral discs lack vascular supply, and the extracellular matrix (ECM) is extremely dense, constituting a natural pharmacokinetic and mass transport barrier that severely limits effective accumulation of therapeutic molecules at target sites [168]. Overcoming this delivery bottleneck has become a core consideration in next‐generation orthopedic drug design [169]: small molecules such as SS‐31 exploit the tissue penetration advantage conferred by their optimized aromatic‐cationic structure, whereas macromolecular strategies such as PROTACs (molecular weight often exceeding 700 Da, complex spatial conformation) face far more severe challenges in traversing the dense ECM [170], and their intra‐articular delivery efficiency and pharmacokinetic behavior still require systematic optimization.

The molecular and cellular heterogeneity of the diseases constitutes the second translational obstacle. Degenerative musculoskeletal diseases are by no means homogeneous pathological processes: whether between individuals or within the local lesion microenvironment of the same patient, the molecular mechanisms and cellular phenotypes driving MQC network imbalance differ significantly [10], which is precisely the root cause of the repeated failure of traditional “one‐size‐fits‐all” intervention strategies.

Inter‐patient heterogeneity manifests as degenerative processes driven by strikingly different dominant pathological axes: some patients are characterized by strong innate immune inflammatory activation (dominated by the cGAS‐STING cascade triggered by mtDNA leakage) [171], whereas others are characterized by cellular energy failure (systematic downregulation of the PGC‐1α metabolic axis) or irreversible accumulation of dysfunctional senescent cells (dominated by the SASP paracrine network) [172]. This macroscopic pathological difference requires rigorous molecular stratification of patient cohorts before initiating targeted therapy.

Intra‐tissue heterogeneity is equally prominent: even within the same affected joint or disc lesion, cell communities exhibit highly fragmented functional states. As described in Section 4.2, scRNA‐seq has revealed highly specific “mitochondrial regulatory chondrocyte” subpopulations lurking in severely degenerated cartilage matrix, and spatial metabolomics has confirmed co‐localization of “lactate hotspots” and “succinate islands” with severely degraded matrix zones [173]. Current evidence suggests that macroscopic tissue degeneration is driven to a large extent by these “pathological hub” cells, which are scattered within relatively healthy tissue and under extreme metabolic stress; therefore, the success of MQC‐targeted therapy will depend to a large extent on the ability to sensitively capture and decode these pathological subpopulations, which imposes explicit requirements on next‐generation precision diagnostic systems (see Section 4.3 subtyping framework).

### 5.4 Support System: Precision Stratification, Biomimetic Models, and Intelligent Decision‐Making

To address the biological delivery barriers and microenvironmental heterogeneity described above, a three‐dimensional support system encompassing precision diagnostic stratification, high‐fidelity biomimetic models, and data‐driven intelligent decision‐making must be constructed [100]; it serves as the central link between innovative intervention strategies and clinical success.

#### 5.4.1 Precision Diagnosis and Patient Stratification

The prerequisite for implementing precision medicine is the development of diagnostic tools that can sensitively capture disease‐driving heterogeneity and precisely classify patients into molecular subtypes highly responsive to specific interventions [174]. The ccf‐mtDNA liquid biopsy (Section 4.1) and multi‐omics molecular subtyping (Sections 4.2‐4.3) together constitute an MQC subtyping diagnostic chain [175]: the former provides real‐time quantitative monitoring of mitochondrial damage, while the latter defines three molecular subtypes at the single‐cell level: bioenergetic failure, immuno‐inflammatory activation, and senescent cell accumulation. The supporting evidence and validation routes are described in Section 4.3.4.

#### 5.4.2 Biomimetic Models: Organ‐on‐a‐Chip as an Accelerator for Translational Validation

Traditional two‐dimensional plastic culture dishes and animal models face insurmountable species and spatial limitations in faithfully recapitulating the complex three‐dimensional microenvironment and dynamic pathological processes of the human musculoskeletal system [176]; microfluidics‐based organ‐on‐a‐chip technology provides a high‐fidelity in vitro validation platform to bridge the translational gap [177]. Bone‐on‐a‐chip (BoC) and joint‐on‐a‐chip (JoC) platforms can integrate fluid shear stress, cyclic mechanical compression, and tissue‐specific oxygen gradients (such as the extreme hypoxia of the nucleus pulposus) [178], recapitulating the key MQC‐regulating signals experienced by skeletal tissue. Using JoC platforms, researchers can observe in real time how accumulation of mitochondrial metabolites (such as lactate) induces epigenetic alterations in neighboring cells (such as H3K18la histone lactylation) [179], providing an ideal pathological simulation environment for screening novel drugs that block “metabolic‐epigenetic” crosstalk; chip‐embedded biosensors can non‐invasively, continuously, and in real time monitor core parameters such as mitochondrial membrane potential fluctuations and ROS bursts [180], thereby enabling efficient mechanistic validation and toxicity assessment of macromolecules such as PTM modulators or PROTACs under near‐physiological conditions, significantly improving the precision of drug toxicological evaluation.

#### 5.4.3 Intelligent Decision‐Making: Toward Data‐Driven Closed‐Loop R&D

Confronted with exponentially growing multi‐omics data, extremely complex in vitro disease models, and increasingly diverse targeted therapeutic options, artificial intelligence and computational biology are rapidly emerging as the “central brain” for integrating massive information and optimizing clinical decision‐making [154]. Constructing “digital twins” and predictive models: by deeply integrating individual whole‐genome sequencing, multi‐omics feature sets, and real‐world clinical indicators, patient‐ or disease‐subtype‐specific “digital twins” can be constructed [181], dynamically simulating disease physiological progression and real‐time MQC network interaction states, enabling large‐scale virtual drug screening in digital space and pre‐computing the perturbation effects of complex intervention strategies such as multi‐target combinations on specific molecular subtypes, precisely anchoring the most translationally promising therapeutic regimens [182]. Optimizing clinical trials and dynamic treatment pathways: advanced machine learning algorithms can precisely extract, from high‐dimensional complex biomarker matrices, the patient features most likely to respond to specific targeted therapies, guiding enrichment trial design and fundamentally improving the success rate of novel drug development [183]; through continuous ingestion of real‐world evidence (RWE) and longitudinal multi‐omics follow‐up data, intelligent systems can iteratively self‐improve and dynamically recommend optimal combination treatment sequences or individualized dose adjustment regimens to clinicians, ultimately constructing a closed‐loop health management ecosystem of “precision diagnosis‐targeted therapy‐real‐time monitoring‐algorithmic optimization”. The clinical value of digital twins depends on data quality and external validation, and they currently remain at the conceptual exploration stage.

Precision stratification, biomimetic models, and computational intelligence together form a stepwise drug translation pipeline (Figure 3): diagnostic tools first locate the correct therapeutic targets and beneficiary subpopulations in complex populations; high‐fidelity microfluidic platforms then assess the efficacy and toxicity of targeted strategies in near‐real biological contexts; and the AI brain finally designs optimal individualized dosing routes and trial paradigms from a global perspective. The integration of this multidimensional support system is likely to be required for bridging the translational gap and moving mitochondrial‐targeted therapies from basic theory to real clinical application.

**FIGURE 3:**
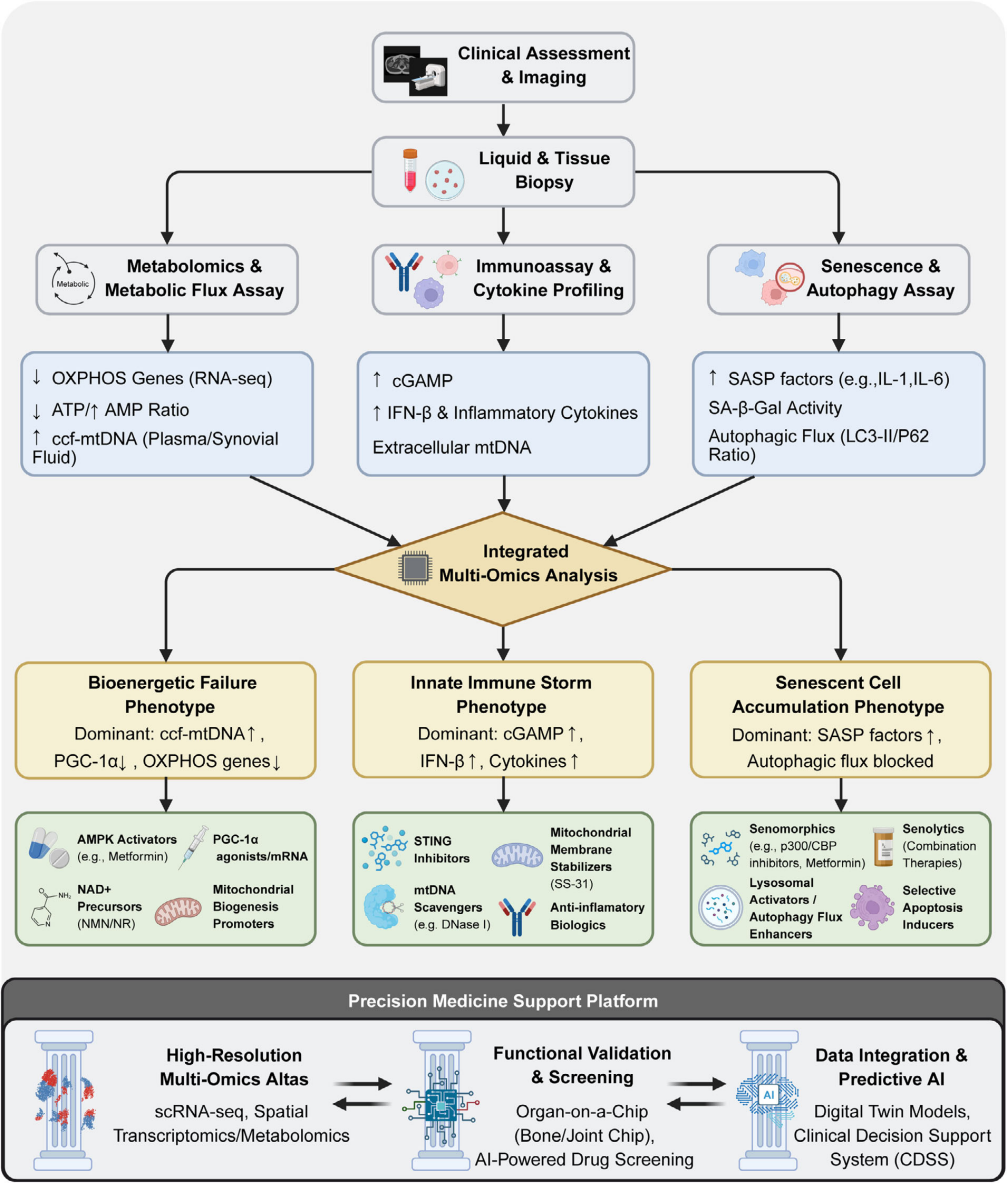
Precision medicine workflow for degenerative musculoskeletal diseases from diagnosis to targeted intervention. Multi‐omics analysis stratifies patients into different mitochondrial phenotypes to guide individualized treatment. **(Upper)** Diagnosis and multi‐omics profiling: the workflow begins with clinical imaging suspicion, followed by liquid and tissue biopsy; samples are analyzed through three parallel omics routes: metabolomics (assessing energy metabolism status, such as ATP/AMP ratio), immune profiling (assessing inflammatory mediators), and senescence/autophagy profiling (assessing SASP factors and autophagic flux). **(Middle)** Molecular subtyping decision: integrated multi‐omics data divide patients into three molecular subtypes: “bioenergetic failure” characterized by energy deficiency, “immuno‐inflammatory activation” marked by elevated cGAMP/IFN‐β, and “senescent cell accumulation” characterized by elevated SASP and obstructed autophagy. **(Lower)** Targeted intervention and support platform: matching intervention strategies are selected based on stratification results, such as NAD^+^ precursors for the bioenergetic failure subtype, STING inhibitors for the immuno‐inflammatory subtype, and senolytic therapy for the senescent cell accumulation subtype; the entire workflow is supported by the precision medicine platform at the bottom, integrating high‐resolution atlases, organoid functional screening, and AI prediction models.

## 6 Conclusions

Collapse of the MQC network provides a common operational thread through the three degenerative musculoskeletal diseases. Mitochondrial dynamics, mitophagy‐biogenesis coupling, and inter‐organelle communication converge on mtDNA release and metabolic intermediate accumulation. These events sustain sterile immune inflammation through mtDNA‐driven DAMPs and reshape the epigenetic landscape. Whether they together create a durable “metabolic memory” in joint and bone tissue, and how much this memory contributes to the apparent irreversibility of degeneration, remain open questions that current evidence supports but does not yet prove (Section 3.3).

In the diagnostic dimension, a chain centered on ccf‐mtDNA liquid biopsy and multi‐omics molecular subtyping may stratify patients into three MQC subtypes: bioenergetic failure, immuno‐inflammatory activation, and senescent cell accumulation. This framework is a conceptual prototype: its stability within OA and its ability to predict outcomes await prospective validation in cohorts such as OAI and MOST, whereas its generalizability to IVDD and OP requires dedicated cross‐disease comparisons with identical analytical pipelines (Section 4.3.4). Therapeutically, evidence levels differ substantially: metformin has the strongest randomized evidence in a musculoskeletal population, and NAD^+^ precursors have entered early‐phase human studies, whereas SS‐31 has received accelerated approval in Barth syndrome while joint‐field data remain insufficient, senolytics have pivoted after the UBX0101 failure, and PROTACs, DdCBE, and mitochondrial transplantation remain preclinical (Table 3). Near‐term efforts should therefore prioritize prospective validation of the most evidence‐backed strategies and subtyping biomarkers; medium‐term goals include sequential and combination strategies and delivery breakthroughs; and the long‐term vision rests on base editing, mitochondrial transplantation, and digital twins. Restoring MQC homeostasis is a central therapeutic goal, but its clinical realization must be premised on evidence grading and honest translational assessment (Figure 4).

**FIGURE 4:**
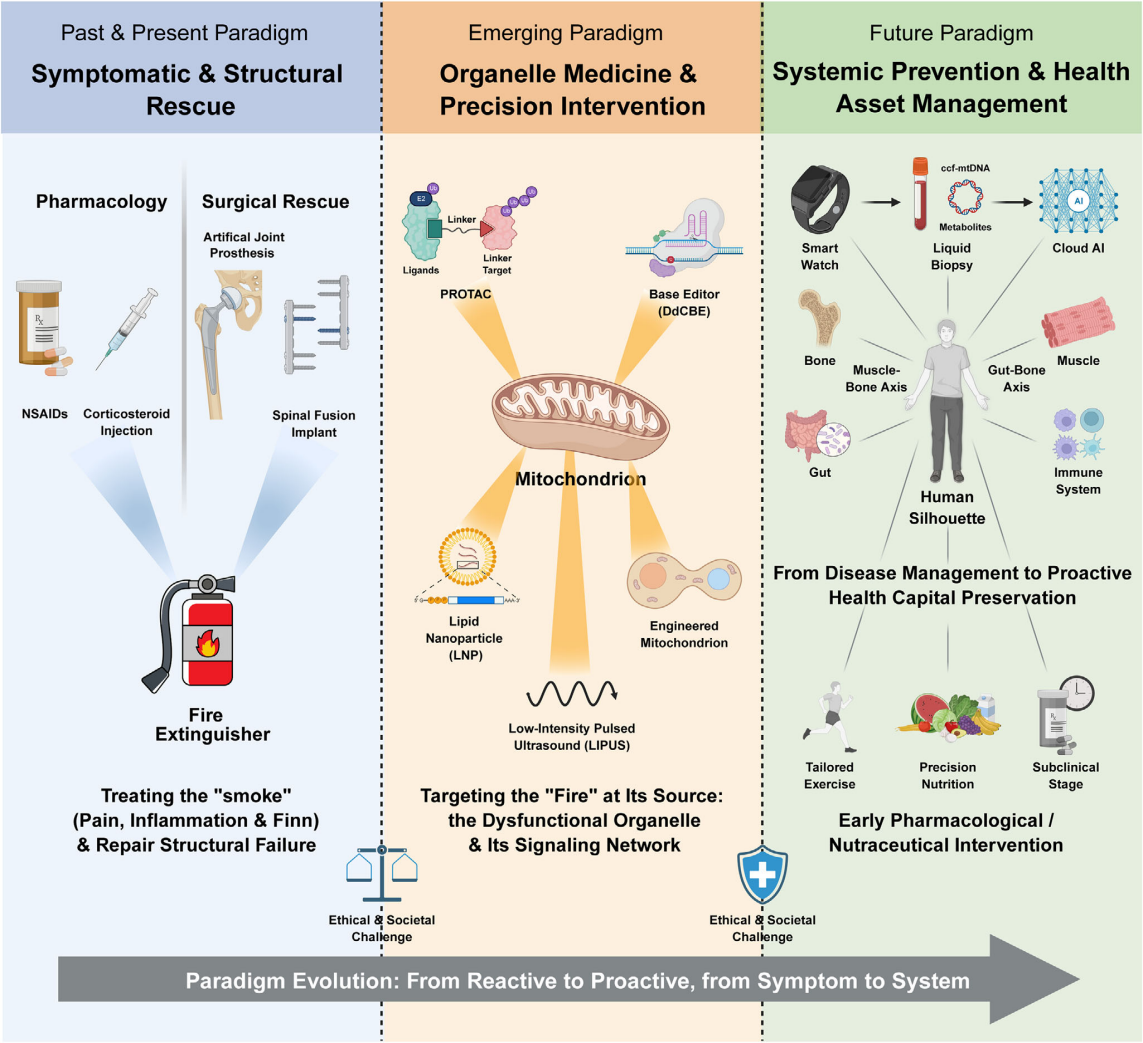
From symptomatic treatment to systematic prevention: the evolution of therapeutic paradigms for degenerative musculoskeletal diseases. Musculoskeletal disease management strategies have evolved through three stages: past/present, emerging, and future. **(Left)** Past and present paradigm (symptom management and structural salvage): traditional treatment models focus on symptom management and end‐stage structural salvage surgery, essentially “treating the symptoms without addressing the root cause”. **(Middle)** Emerging paradigm (organelle medicine and precision intervention): focusing on the disease root cause (dysfunctional mitochondria), precision tools including PROTACs, base editors, lipid nanoparticle (LNP) delivery systems, and engineered mitochondrial transplantation are employed to restore organelle health. **(Right)** Future paradigm (systemic prevention and proactive health management): emphasizing prospective, holistic health maintenance through real‐time monitoring of systemic indicators with wearable devices and liquid biopsy, combined with cloud‐based AI analysis, to implement individualized lifestyle interventions (tailored exercise, precision nutrition) and pharmacological interventions at early subclinical stages. The large arrow at the bottom summarizes this evolutionary trajectory: from passive, symptom‐driven reactive treatment toward proactive, systems‐biology‐driven preventive health management, while also indicating the ethical and social challenges that must be addressed throughout the process.

## 7 Future Perspectives

Looking ahead, the nearest‐term prospect is systems biology‐driven intelligent closed‐loop R&D: patient‐specific “digital twin” models built from multi‐scale omics data, whose limitations are discussed in Section 5.4.3, combined with high‐throughput validation on microfluidic biomimetic chips, would close the chain from individualized risk prediction to precision intervention, provided that external validation of prediction accuracy is first completed in a single disease such as knee OA. Cross‐organ network medicine represents the medium‐term direction: embedding the skeletal system within the systemic immune‐metabolic network and using MQC‐targeting drugs to improve muscle, bone, and metabolic organ function simultaneously, a strategy for which causal evidence is still confined to animal models and requires interdisciplinary validation, and whose two leading candidates (metformin, NAD+ precursors) currently rest on different evidence levels (Section 5.2). This direction is already taking shape elsewhere: MQC‐targeted strategies have reached an advanced stage in cardiovascular medicine [184], showing that organelle‐level mechanisms can be translated in a major organ system, although skeletal application will still require organ‐specific validation. The long‐term vision is a proactive health ecosystem centered on early intervention: wearable biosensing and high‐frequency dynamic liquid biopsy would enable longitudinal risk monitoring and individualized intervention in the subclinical stage, moving the therapeutic threshold before disease onset.

These projections remain conditional: they depend on prospective validation of the causal relationships between subtyping biomarkers and intervention strategies, and on resolution of delivery and safety obstacles. The accompanying ethical and social challenges require forward‐looking responses: clinical application of mitochondrial base editing [185] must be placed under rigorous ethical review and regulatory frameworks, and functional organelle transplantation will require a comparable framework as it approaches the clinic. Data privacy, algorithmic fairness, and healthcare equity raise separate questions for the AI‐driven decision layer discussed in Section 5.4.3. By redefining skeletal health as a dynamic output of systemic homeostasis, orthopedics may move beyond delaying tissue damage toward maintaining skeletal vitality throughout the life course.

## Author Contributions

**Haiyang Gao**: Conceptualization, Investigation, Methodology, Visualization, Writing – original draft; **Zhangrong Cheng**: Conceptualization, Investigation, Methodology, Visualization, Writing – original draft; **Xianglong Chen**: Validation, Formal analysis; **Wenbo Wu**: Investigation, Validation; **Wang Wu**: Validation, Formal analysis; **Zimu Yu**: Validation, Formal analysis; **Cao Yang**: Supervision, Writing – review & editing; **Yukun Zhang**: Conceptualization, Supervision, Funding acquisition, Writing – review & editing.

## Funding

This work was supported by the National Natural Science Foundation of China (No. 82172497) and China Postdoctoral Science Foundation (NO. 2025M782003).

## Conflicts of Interest

The authors declare no conflicts of interest.

## Data Availability

Data sharing not applicable to this article as no datasets were generated or analysed during the current study.

## References

[1] N. Fine , S. Lively , C. A. Séguin , A. V. Perruccio , M. Kapoor , and R. Rampersaud , “Intervertebral Disc Degeneration And Osteoarthritis: A Common Molecular Disease Spectrum,” Nature Reviews Rheumatology 19 (2023): 136–152. 10.1038/s41584-022-00888-z 36702892

[2] G. Wei , K. Lu , M. Umar , et al., “Risk of Metabolic Abnormalities In Osteoarthritis: A New Perspective To Understand Its Pathological Mechanisms,” Bone Research 11 (2023): 63. 10.1038/s41413-023-00301-9 PMC10698167 38052778

[3] M. Favero , E. Belluzzi , A. Ortolan , et al., “Erosive Hand Osteoarthritis: Latest Findings And Outlook,” Nature Reviews Rheumatology 18 (2022): 171–183. 10.1038/s41584-021-00747-3 35105980

[4] M. Schieker , P. G. Conaghan , L. Mindeholm , et al., “Effects of Interleukin‐1β Inhibition on Incident Hip and Knee Replacement: Exploratory Analyses From a Randomized, Double‐Blind, Placebo‐Controlled Trial,” Annals of Internal Medicine 173 (2020): 509–515. 10.7326/M20-0527 PMC8503784 32744862

[5] K. H. Collins , I. K. Haugen , T. Neogi , and F. Guilak , “Osteoarthritis as a Systemic Disease,” Nature Reviews Rheumatology 22 (2026): 105–117. 10.1038/s41584-025-01332-8 PMC13556116 41339496

[6] W. Jiang , H. Liu , R. Wan , Y. Wu , Z. Shi , and W. Huang , “Mechanisms Linking Mitochondrial Mechanotransduction And Chondrocyte Biology In The Pathogenesis Of Osteoarthritis,” Ageing Research Reviews 67 (2021): 101315. 10.1016/j.arr.2021.101315 33684550

[7] T. Wang , L. Wang , L. Zhang , Y. Long , Y. Zhang , and Z. Hou , “Single‐Cell RNA Sequencing in Orthopedic Research,” Bone Research 11 (2023): 10. 10.1038/s41413-023-00245-0 PMC9958119 36828839

[8] Q. Yao , X. Wu , C. Tao , et al., “Osteoarthritis: Pathogenic Signaling Pathways And Therapeutic Targets,” Signal Transduct Target Ther 8 (2023): 56. 10.1038/s41392-023-01330-w PMC9898571 36737426

[9] A. Mahmoudian , L. S. Lohmander , A. Mobasheri , M. Englund , and F. P. Luyten , “Early‐Stage Symptomatic Osteoarthritis Of The Knee – Time For Action,” Nature Reviews Rheumatology 17 (2021): 621–632. 10.1038/s41584-021-00673-4 34465902

[10] E. Sanchez‐Lopez , R. Coras , A. Torres , N. E. Lane , and M. Guma , “Synovial Inflammation In Osteoarthritis Progression,” Nature Reviews Rheumatology 18 (2022): 258–275. 10.1038/s41584-022-00749-9 PMC9050956 35165404

[11] Y. Song , S. Lu , W. Geng , et al., “Mitochondrial Quality Control In Intervertebral Disc Degeneration,” Experimental & Molecular Medicine 53 (2021): 1124–1133. 10.1038/s12276-021-00650-7 PMC8333068 34272472

[12] K. Sun , X. Jing , J. Guo , X. Yao , and F. Guo , “Mitophagy in Degenerative Joint Diseases,” Autophagy 17 (2021): 2082–2092. 10.1080/15548627.2020.1822097 PMC8496714 32967533

[13] A. Larrañaga‐SanMiguel , N. Bengoa‐Vergniory , and H. Flores‐Romero , “Crosstalk Between Mitochondria‐Er Contact Sites And The Apoptotic Machinery As A Novel Health Meter,” Trends in Cell Biology 35 (2025): 33–45. 10.1016/j.tcb.2024.08.007 39379268

[14] J. Cai , Z. Chen , X. Yang , et al., “Role of Mitochondrial Damage‐Associated Molecular Patterns In Osteoarthritis: From Pathogenesis, Diagnosis, And Prognosis To Therapeutics,” Pharmacol Res 219 (2025): 107865. 10.1016/j.phrs.2025.107865 40712761

[15] D. Liu , Z.‐J. Cai , Y.‐T. Yang , et al., “Mitochondrial Quality Control In Cartilage Damage And Osteoarthritis: New Insights And Potential Therapeutic Targets,” Osteoarthritis and Cartilage 30 (2022): 395–405. 10.1016/j.joca.2021.10.009 34715366

[16] B. Qiu , X. Xie , and Y. Xi , “Mitochondrial Quality Control: The Real Dawn Of Intervertebral Disc Degeneration?,” Journal of Translational Medicine 22 (2024): 1126. 10.1186/s12967-024-05943-9 PMC11662537 39707402

[17] F. den Brave , U. Schulte , and B. Fakler , “Mitochondrial Complexome and Import Network,” Trends in Cell Biology 34 (2024): 578–594. 10.1016/j.tcb.2023.10.004 37914576

[18] L. C. Tábara , M. Segawa , and J. Prudent , “Molecular Mechanisms of Mitochondrial Dynamics,” Nature Reviews Molecular Cell Biology 26 (2025): 123–146. 10.1038/s41580-024-00785-1 39420231

[19] O. C. Losón , Z. Song , H. Chen , and D. C. Chan , “Fis1, Mff, MiD49, and MiD51 mediate Drp1 Recruitment In Mitochondrial Fission,” Molecular Biology of the Cell 24 (2013): 659–667. 10.1091/mbc.E12-10-0721 PMC3583668 23283981

[20] Z. Qin , X. Zhu , Y. Shen , et al., “An Emerging Role Of Mitochondrial Quality Control In Bone Metabolism: From Molecular Mechanisms To Targeted Therapeutic Interventions,” Cellular and Molecular Life Sciences 82 (2025): 291. 10.1007/s00018-025-05802-w PMC12307276 40728722

[21] A. Jahani‐Asl and R. S. Slack , “The phosphorylation state of Drp1 determines cell fate,” EMBO Reports 8 (2007): 912–913. 10.1038/sj.embor.7401077 PMC2002565 17906671

[22] R. Seager , N. S. Ramesh , S. Cross , C. Guo , K. A. Wilkinson , and J. M. Henley , “SUMOylation of MFF Coordinates Fission Complexes To Promote Stress‐Induced Mitochondrial Fragmentation,” Science Advance 10 (2024): adq6223. 10.1126/sciadv.adq6223 PMC11451547 39365854

[23] Z. Lin , X. Lu , G. Xu , et al., “The Mitochondrial E3 Ligase MAPL SUMOylates Drp1 to Facilitate Mitochondrial Fission In Intervertebral Disc Degeneration,” Bone Research 13 (2025): 72. 10.1038/s41413-025-00449-6 PMC12343876 40796734

[24] P. Mishra , V. Carelli , G. Manfredi , and D. C. Chan , “Proteolytic Cleavage of Opa1 Stimulates Mitochondrial Inner Membrane Fusion And Couples Fusion To Oxidative Phosphorylation,” Cell Metabolism 19 (2014): 630–641. 10.1016/j.cmet.2014.03.011 PMC4018240 24703695

[25] Y. Deng , M. Hou , Y. Wu , et al., “SIRT3‐PINK1‐PKM2 Axis Prevents Osteoarthritis Via Mitochondrial Renewal And Metabolic Switch,” Bone Research 13 (2025): 36. 10.1038/s41413-025-00413-4 PMC11909255 40087281

[26] C. Y. Kuo , C. Shieh , F. Cai , et al., “Coordinate to Guard: Crosstalk Of Phosphorylation, Sumoylation, And Ubiquitylation in DNA Damage Response,” Frontiers in Oncology 1 (2011): 61. 10.3389/fonc.2011.00061 PMC3355946 22649771

[27] K. Palikaras , E. Lionaki , and N. Tavernarakis , “Mechanisms of Mitophagy In Cellular Homeostasis, Physiology And Pathology,” Nature Cell Biology 20 (2018): 1013–1022. 10.1038/s41556-018-0176-2 30154567

[28] O. Daumke and M. van der Laan , “Molecular Machineries Shaping The Mitochondrial Inner Membrane,” Nature Reviews Molecular Cell Biology 26 (2025): 706–724. 10.1038/s41580-025-00854-z 40369159

[29] D. P. Narendra and R. J. Youle , “The role of PINK1‐Parkin in Mitochondrial Quality Control,” Nature Cell Biology 26 (2024): 1639–1651. 10.1038/s41556-024-01513-9 39358449

[30] Y. Chen and G. W. Dorn II , “PINK1‐Phosphorylated Mitofusin 2 is a Parkin Receptor for Culling Damaged Mitochondria,” Science 340 (2013): 471–475. 10.1126/science.1231031 PMC3774525 23620051

[31] M. Lazarou , D. A. Sliter , L. A. Kane , et al., “The Ubiquitin Kinase PINK1 Recruits Autophagy Receptors to Induce Mitophagy,” Nature 524 (2015): 309–314. 10.1038/nature14893 PMC5018156 26266977

[32] V. Basso , E. Marchesan , C. Peggion , et al., “Regulation of ER‐Mitochondria Contacts by Parkin via Mfn2,” Pharmacol Res 138 (2018): 43–56. 10.1016/j.phrs.2018.09.006 30219582

[33] M. Rybarski , D. Mrohs , K. Osenberg , et al., “Loss of Parkin Causes Endoplasmic Reticulum Calcium Dyshomeostasis By Upregulation Of Reticulocalbin 1,” The European journal of Neuroscience 57 (2023): 739–761. 10.1111/ejn.15917 36656174

[34] S.‐Y. Lee , H.‐J. An , J. M. Kim , et al., “PINK1 Deficiency Impairs Osteoblast Differentiation Through Aberrant Mitochondrial Homeostasis,” Stem Cell Res Ther 12 (2021): 589. 10.1186/s13287-021-02656-4 PMC8614054 34823575

[35] R. C. Scarpulla , R. B. Vega , and D. P. Kelly , “Transcriptional Integration Of Mitochondrial Biogenesis,” Trends in Endocrinology & Metabolism 23 (2012): 459–466. 10.1016/j.tem.2012.06.006 PMC3580164 22817841

[36] Z. Wu , P. Puigserver , U. Andersson , et al., “Mechanisms Controlling Mitochondrial Biogenesis And Respiration Through The Thermogenic Coactivator PGC‐1,” Cell 98 (1999): 115–124. 10.1016/S0092-8674(00)80611-X 10412986

[37] H. Wang , J. Su , M. Yu , Y. Xia , and Y. Wei , “PGC‐1α in Osteoarthritic Chondrocytes: From Mechanism To Target Of Action,” Frontiers in Pharmacology 14 (2023): 1169019. 10.3389/fphar.2023.1169019 PMC10117990 37089944

[38] J. C. Casler , C. S. Harper , A. J. White , H. L. Anderson , and L. L. Lackner , “Mitochondria‐ER‐PM contacts regulate mitochondrial division and PI(4)P distribution,” Journal of Cell Biology 223 (2024): e202308144. 10.1083/jcb.202308144 PMC11116812 38781029

[39] M. R. Wozny , A. Di Luca , D. R. Morado , et al., “In situ architecture of the ER‐mitochondria encounter structure,” Nature 618 (2023): 188–192. 10.1038/s41586-023-06050-3 PMC7614606 37165187

[40] S. Nagashima , L.‐C. Tábara , L. Tilokani , et al., “Golgi‐Derived PI(4)P‐Containing Vesicles Drive Late Steps Of Mitochondrial Division,” Science 367 (2020): 1366–1371. 10.1126/science.aax6089 32193326

[41] Y. Lu , J. Zhou , H. Wang , et al., “Endoplasmic Reticulum Stress‐Mediated Apoptosis And Autophagy In Osteoarthritis: From Molecular Mechanisms To Therapeutic Applications,” Cell Stress Chaperones 29 (2024): 805–830. 10.1016/j.cstres.2024.11.005 PMC11667160 39571722

[42] X. Ma , S. Manley , H. Qian , et al., “Mitochondria‐Lysosome‐Related Organelles Mediate Mitochondrial Clearance During Cellular Dedifferentiation,” Cell Reports 42 (2023): 113291. 10.1016/j.celrep.2023.113291 PMC10842364 37862166

[43] J. E. Kim , S.‐Y. Park , C. Kwak , et al., “Glucose‐Mediated Mitochondrial Reprogramming By Cholesterol Export at TM4SF5‐Enriched Mitochondria‐Lysosome Contact Sites,” Cancer Commun (Lond) 44 (2024): 47–75. 10.1002/cac2.12510 PMC10794009 38133457

[44] C. Settembre , C. Di Malta , V. A. Polito , et al., “TFEB Links Autophagy To Lysosomal Biogenesis,” Science 332 (2011): 1429–1433. 10.1126/science.1204592 PMC3638014 21617040

[45] M. Iavazzo , L. Cinque , S. Levantovsky , et al., “TFEB Coordinates Autophagosome Biogenesis And Ribophagy During Starvation Via SQSTM1,” Science Advance 12 (2026): aea9302. 10.1126/sciadv.aea9302 PMC12757066 41477847

[46] S. Kang , S. Liu , X. Dong , et al., “USP4 Depletion‐Driven RAB7A Ubiquitylation Impairs Autophagosome‐Lysosome Fusion And Aggravates Periodontitis,” Autophagy 21 (2025): 771–788. 10.1080/15548627.2024.2429371 PMC11925113 39663592

[47] Y. Rong , S. Zhang , N. Nandi , et al., “STING Controls Energy Stress‐Induced Autophagy And Energy Metabolism Via STX17,” Journal of Cell Biology 221 (2022): e202202060. 10.1083/jcb.202202060 PMC9082627 35510944

[48] S. Liu , Y. Hu , W. Xu , et al., “Restoration of Lysosomal Function Attenuates Autophagic Flux Impairment In Nucleus Pulposus Cells And Protects Against Mechanical Overloading‐Induced Intervertebral Disc Degeneration,” Autophagy 21 (2025): 979–995. 10.1080/15548627.2024.2440844 PMC12013417 39675125

[49] S. Marchi , E. Guilbaud , S. W. G. Tait , T. Yamazaki , and L. Galluzzi , “Mitochondrial control of inflammation,” Nature Reviews Immunology 23 (2023): 159–173. 10.1038/s41577-022-00760-x PMC9310369 35879417

[50] J. S. Riley and S. W. Tait , “Mitochondrial DNA in Inflammation and Immunity,” EMBO Reports 21 (2020): 49799. 10.15252/embr.201949799 PMC7132203 32202065

[51] W. Zhang , G. Li , R. Luo , et al., “Cytosolic escape of mitochondrial DNA triggers cGAS‐STING‐NLRP3 Axis‐Dependent Nucleus Pulposus Cell Pyroptosis,” Experimental & Molecular Medicine 54 (2022): 129–142. 10.1038/s12276-022-00729-9 PMC8894389 35145201

[52] P. Mishra and D. C. Chan , “Mitochondrial Dynamics And Inheritance During Cell Division, Development And Disease,” Nature Reviews Molecular Cell Biology 15 (2014): 634–646. 10.1038/nrm3877 PMC4250044 25237825

[53] M. Giacomello , A. Pyakurel , C. Glytsou , and L. Scorrano , “The Cell Biology Of Mitochondrial Membrane Dynamics,” Nature Reviews Molecular Cell Biology 21 (2020): 204–224. 10.1038/s41580-020-0210-7 32071438

[54] J. Geiger , F. Klötzer , N. Pollak , G. Fullstone , and M. Rehm , “Stochasticity Contributes to Explaining Minority and Majority MOMP During Apoptosis,” Cell Death Disease 16 (2025): 893. 10.1038/s41419-025-08258-9 PMC12717247 41419664

[55] J. Kim , R. Gupta , L. P. Blanco , et al., “VDAC Oligomers form Mitochondrial Pores to Release mtDNA Fragments and Promote Lupus‐Like Disease,” Science 366 (2019): 1531–1536. 10.1126/science.aav4011 PMC8325171 31857488

[56] M. Motwani , S. Pesiridis , and K. A. Fitzgerald , “DNA Sensing by the cGAS‐STING Pathway in Health and Disease,” Nature Reviews Genetics 20 (2019): 657–674. 10.1038/s41576-019-0151-1 31358977

[57] Z. Gao , Z. Gao , H. Zhang , S. Hou , Y. Zhou , and X. Liu , “Targeting STING: From antiviral immunity to treat osteoporosis,” Front Immunol 13 (2022): 1095577. 10.3389/fimmu.2022.1095577 PMC9891206 36741390

[58] S. Victorelli , H. Salmonowicz , J. Chapman , et al., “Apoptotic Stress Causes mtDNA Release During Senescence and Drives the SASP,” Nature 622 (2023): 627–636. 10.1038/s41586-023-06621-4 PMC10584674 37821702

[59] K. Hu , Y. Lan , T. Song , Y. Song , and M. Song , “Revisiting Osteoarthritis Pathogenesis Through The Lens of cGAS‐STING: Mitochondrial Damage, Pyroptosis, And Inflammatory Cascades,” Journal of Orthopaedic Translation 56 (2026): 101010. 10.1016/j.jot.2025.10.002 PMC12988517 41836584

[60] A. Decout , J. D. Katz , S. Venkatraman , and A. Ablasser , “The cGAS‐STING Pathway As A Therapeutic Target In Inflammatory Diseases,” Nature Reviews Immunology 21 (2021): 548–569. 10.1038/s41577-021-00524-z PMC8029610 33833439

[61] D. G. Ryan and L. A. J. O'Neill , “Krebs Cycle Rewired For Macrophage And Dendritic Cell Effector Functions,” FEBS Letters 591 (2017): 2992–3006. 10.1002/1873-3468.12744 28685841

[62] S. Culemann , A. Grüneboom , J. Á. Nicolás‐Ávila , et al., “Locally Renewing Resident Synovial Macrophages Provide A Protective Barrier For The Joint,” Nature 572 (2019): 670–675. 10.1038/s41586-019-1471-1 PMC6805223 31391580

[63] N. T. Sprenkle and E. L. Mills , “Metabolite and Nutrient Regulation Of Macrophages In Obesity And Metabolic Disease,” Nature Reviews Immunology 26 (2026): 613–626. 10.1038/s41577-026-01292-4 41986489

[64] L. Zheng , Z. Zhang , P. Sheng , and A. Mobasheri , “The Role Of Metabolism In Chondrocyte Dysfunction And The Progression Of Osteoarthritis,” Ageing Research Reviews 66 (2021): 101249. 10.1016/j.arr.2020.101249 33383189

[65] D. Zhang , Z. Tang , H. Huang , et al., “Metabolic Regulation Of Gene Expression By Histone Lactylation,” Nature 574 (2019): 575–580. 10.1038/s41586-019-1678-1 PMC6818755 31645732

[66] W. Lan , X. Chen , H. Yu , et al., “UGDH Lactylation Aggravates Osteoarthritis by Suppressing Glycosaminoglycan Synthesis and Orchestrating Nucleocytoplasmic Transport to Activate MAPK Signaling,” Advance Science 12 (2025): 2413709. 10.1002/advs.202413709 PMC12120796 40150862

[67] F. Lu , Y. Yu , G. Yin , et al., “FSTL1 Orchestrates Metabolic‐Epigenetic Crosstalk: Glycolysis‐Dependent H3K18 Lactylation Drives Cartilage Fibrosis in Osteoarthritis,” Advance Science (Weinh) 13 (2026): 12002. 10.1002/advs.202512002 PMC12884748 41293926

[68] M. Giacomello and L. Pellegrini , “The Coming Of Age Of The Mitochondria‐ER Contact: A Matter of Thickness,” Cell Death Differentiation 23 (2016): 1417–1427. 10.1038/cdd.2016.52 PMC5072433 27341186

[69] M. Hou , Y. Ma , Y. Deng , et al., “Therapeutic Restoration Of Mitochondria‐Endoplasmic Reticulum Cross Talk For Osteoarthritis,” The Proceedings of the National Academy of Sciences 122 (2025): 2426992122. 10.1073/pnas.2426992122 PMC12435279 40892916

[70] O. M. de Brito and L. Scorrano , “Mitofusin 2 Tethers Endoplasmic Reticulum To Mitochondria,” Nature 456 (2008): 605–610. 10.1038/nature07534 19052620

[71] R. L. Wiseman , J. S. Mesgarzadeh , and L. M. Hendershot , “Reshaping Endoplasmic Reticulum Quality Control Through The Unfolded Protein Response,” Molecular Cell 82 (2022): 1477–1491. 10.1016/j.molcel.2022.03.025 PMC9038009 35452616

[72] E. L. Mills , B. Kelly , A. Logan , et al., “Succinate Dehydrogenase Supports Metabolic Repurposing of Mitochondria to Drive Inflammatory Macrophages,” Cell 167 (2016): 457–470.e13. 10.1016/j.cell.2016.08.064 PMC5863951 27667687

[73] X. A. Zhang and H. Kong , “Mechanism of HIFs in osteoarthritis,” Front Immunol 14 (2023): 1168799. 10.3389/fimmu.2023.1168799 PMC10067622 37020556

[74] M. Xiao , H. Yang , W. Xu , et al., “Inhibition of α‐KG‐Dependent Histone and DNA Demethylases By Fumarate And Succinate That Are Accumulated In Mutations of FH and SDH Tumor Suppressors,” Genes and Development 26 (2012): 1326–1338. 10.1101/gad.191056.112 PMC3387660 22677546

[75] Z. Li , J. Zhang , T. Xu , Z. Hao , and Y. Li , “Mechanism of Histone Demethylase KDM5A in Osteoporotic Fracture Healing Through Epigenetic Regulation of the miR‐495/SKP2/Runx2 axis,” Molecular Medicine 31 (2025): 65. 10.1186/s10020-025-01098-5 PMC11837617 39972431

[76] X. Zhu , Z. Chen , W. Shen , et al., “Inflammation, Epigenetics, And Metabolism Converge To Cell Senescence And Ageing: The Regulation And Intervention,” Signal Transduction Targeted Therapy 6 (2021): 245. 10.1038/s41392-021-00646-9 PMC8236488 34176928

[77] S.‐I. Inoue , M. J. Emmett , H.‐W. Lim , et al., “Short‐Term Cold Exposure Induces Persistent Epigenomic Memory In Brown Fat,” Cell Metabolism 36 (2024): 1764–1778.e9. 10.1016/j.cmet.2024.05.011 PMC11305953 38889724

[78] G. S. Ducker and J. D. Rabinowitz , “One‐Carbon Metabolism in Health and Disease,” Cell Metabolism 25 (2017): 27–42. 10.1016/j.cmet.2016.08.009 PMC5353360 27641100

[79] T. Teglas , F. Torma , Z. Bori , et al., “Epigenetic Insights of Olympic Champions: Nuclear and Mitochondrial DNA Methylation and Regulators of Aging,” Geroscience 48 (2026): 7923–7938. 10.1007/s11357-025-02092-9 PMC13638895 41547678

[80] D. Zhu , X. Li , and Y. Tian , “Mitochondrial‐To‐Nuclear Communication In Aging: An Epigenetic Perspective,” Trends in Biochemical Sciences 47 (2022): 645–659. 10.1016/j.tibs.2022.03.008 35397926

[81] M. T. Yin and L. Guo , “Mitochondrial DNA Methylation: State‐Of‐The‐Art In Molecular Mechanisms And Disease Implications,” Journal of Advanced Research 83 (2026): 455–473. 10.1016/j.jare.2025.08.029 PMC13131407 40848899

[82] S. C. Uzuner , “Mitochondrial DNA Methylation Misleads Global DNA Methylation Detected By Antibody‐Based Methods,” Analytical Biochemistry 601 (2020): 113789. 10.1016/j.ab.2020.113789 32473121

[83] M. Mechta , L. R. Ingerslev , O. Fabre , M. Picard , and R. Barrès , “Evidence Suggesting Absence of Mitochondrial DNA Methylation,” Frontiers in Genetics 8 (2017): 166. 10.3389/fgene.2017.00166 PMC5671948 29163634

[84] S. C. Baksh and L. W. S. Finley , “Metabolic Coordination of Cell Fate by α‐Ketoglutarate‐Dependent Dioxygenases,” Trends in Cell Biology 31 (2021): 24–36. 10.1016/j.tcb.2020.09.010 PMC7748998 33092942

[85] Y. Li , Q. Tu , C. Liu , et al., “The metabolite α‐ketoglutarate Induces AIM2‐Dependent PANoptosis Through Demethylase TET2,” Cell Commun Signal 24 (2026): 193. 10.1186/s12964-026-02740-3 PMC13014779 41699684

[86] A. M. Intlekofer , B. Wang , H. Liu , et al., “L‐2‐Hydroxyglutarate Production Arises From Noncanonical Enzyme Function At Acidic pH,” Nature Chemical Biology 13 (2017): 494–500. 10.1038/nchembio.2307 PMC5516644 28263965

[87] B. Thienpont , J. Steinbacher , H. Zhao , et al., “Tumour Hypoxia Causes DNA Hypermethylation by Reducing TET Activity,” Nature 537 (2016): 63–68. 10.1038/nature19081 PMC5133388 27533040

[88] F. C. Grandi and N. Bhutani , “Epigenetic Therapies for Osteoarthritis,” Trends Pharmacological Sciences 41 (2020): 557–569. 10.1016/j.tips.2020.05.008 PMC10621997 32586653

[89] L. N. Reynard and M. J. Barter , “Osteoarthritis Year in Review 2019: Genetics, Genomics and Epigenetics,” Osteoarthritis and Cartilage 28 (2020): 275–284. 10.1016/j.joca.2019.11.010 31874234

[90] A. Asadi Shahmirzadi , D. Edgar , C.‐Y. Liao , et al., “Alpha‐Ketoglutarate, an Endogenous Metabolite, Extends Lifespan and Compresses Morbidity in Aging Mice,” Cell Metabolism 32 (2020): 447–456.e6. 10.1016/j.cmet.2020.08.004 PMC8508957 32877690

[91] W. Dai , X. Qiao , Y. Fang , et al., “Epigenetics‐Targeted Drugs: Current Paradigms And Future Challenges,” Signal Transduction Targeted Therapy 9 (2024): 332. 10.1038/s41392-024-02039-0 PMC11627502 39592582

[92] C. Huang , Y. Liang , Y. Li , Q. Wei , L. Ouyang , and J. Zhang , “The Epigenetic Landscape Of Rheumatoid Arthritis: Pathogenesis And Drug Therapeutic Potentials,” Acta Pharmaceutica Sinica B 15 (2025): 5601–5631. 10.1016/j.apsb.2025.08.022 PMC12648046 41311406

[93] R. C. Riddle and T. L. Clemens , “Bone Cell Bioenergetics and Skeletal Energy Homeostasis,” Physiological Reviews 97 (2017): 667–698. 10.1152/physrev.00022.2016 PMC5539406 28202599

[94] C. M. Klinge , “Estrogenic Control Of Mitochondrial Function,” Redox Biology 31 (2020): 101435. 10.1016/j.redox.2020.101435 PMC7212490 32001259

[95] K.‐A. Ishii , T. Fumoto , K. Iwai , et al., “Coordination of PGC‐1beta And Iron Uptake In Mitochondrial Biogenesis And Osteoclast Activation,” Nature Medicine 15 (2009): 259–266. 10.1038/nm.1910 19252502

[96] A. N. Smith , F. O. Jouret , S. Bord , et al., “Vacuolar H+‐ATPase d2 Subunit: Molecular Characterization, Developmental Regulation, And Localization To Specialized Proton Pumps In Kidney And Bone,” Journal of the American Society of Nephrology 16 (2005): 1245–1256. 10.1681/ASN.2004090761 15800125

[97] N. K. Lee , Y. G. Choi , J. Y. Baik , et al., “A Crucial Role for Reactive Oxygen Species in RANKL‐Induced Osteoclast Differentiation,” Blood 106 (2005): 852–859. 10.1182/blood-2004-09-3662 15817678

[98] Y. Zhong , Z. Li , H. Hong , et al., “Vertebral BMSC‐EVs Under Estrogen Deficiency Drive Senescence‐Related Mitochondrial Dysfunction In Endplate Chondrocytes via MRPL1 mRNA delivery,” Experimental & Molecular Medicine 58 (2026): 1522–1535. 10.1038/s12276-026-01719-x PMC13234282 42098460

[99] Z. Zhou , T. M. Moore , B. G. Drew , et al., “Estrogen Receptor α Controls Metabolism In White And Brown Adipocytes By Regulating Polg1 and Mitochondrial Remodeling,” Science Translational Medicine 12 (2020): eaax8096. 10.1126/scitranslmed.aax8096 PMC8212422 32759275

[100] D. J. Hunter and S. Bierma‐Zeinstra , “Osteoarthritis,” Lancet 398 (2019): 1745–1759. 10.1016/S0140-6736(19)30417-9 31034380

[101] P. H. L. Putri , S. H. Alamudi , X. Dong , and Y. Fu , “Extracellular Vesicles In Age‐Related Diseases: Disease Pathogenesis, Intervention, And Biomarker,” Stem Cell Research & Therapy 16 (2025): 263. 10.1186/s13287-025-04374-7 PMC12121224 40437603

[102] X. Wu , X. Yang , Y. Dai , et al., “Single‐Cell Sequencing To Multi‐Omics: Technologies And Applications,” Biomarker Research 12 (2024): 110. 10.1186/s40364-024-00643-4 PMC11438019 39334490

[103] Q. Ji , Y. Zheng , G. Zhang , et al., “Single‐cell RNA‐Seq Analysis Reveals The Progression Of Human Osteoarthritis,” Annals of the Rheumatic Diseases 78 (2019): 100–110. 10.1136/annrheumdis-2017-212863 PMC6317448 30026257

[104] C. Trumpff , J. Michelson , C. J. Lagranha , et al., “Stress and Circulating Cell‐Free Mitochondrial DNA: A Systematic Review Of Human Studies, Physiological Considerations, And Technical Recommendations,” Mitochondrion 59 (2021): 225–245. 10.1016/j.mito.2021.04.002 PMC8418815 33839318

[105] J. Xie , Y. Wang , L. Lu , L. Liu , X. Yu , and F. Pei , “Cellular Senescence In Knee Osteoarthritis: Molecular Mechanisms And Therapeutic Implications,” Ageing Research Reviews 70 (2021): 101413. 10.1016/j.arr.2021.101413 34298194

[106] D. J. Klionsky , A. K. Abdel‐Aziz , S. Abdelfatah , et al., “Guidelines for the Use And Interpretation Of Assays For Monitoring Autophagy,” Autophagy 17 (2021): 1–382. 10.1080/15548627.2020.1797280 PMC7996087 33634751

[107] L. Tumburu , S. Ghosh‐Choudhary , F. T. Seifuddin , et al., “Circulating Mitochondrial DNA is a Proinflammatory DAMP in Sickle Cell Disease,” Blood 137 (2021): 3116–3126. 10.1182/blood.2020009063 PMC8176765 33661274

[108] N. Ma , Y. Xu , D. Zhang , et al., “Targeting age‐related cell‐free DNA for Prevention, Early Diagnosis And Treatment Of Rheumatoid Arthritis,” Autoimmun Reviews 25 (2026): 103961. 10.1016/j.autrev.2025.103961 41319827

[109] M. Panagopoulou , M. Karaglani , K. Tzitzikou , et al., “Mitochondrial Fraction of Circulating Cell‐Free DNA as an Indicator of Human Pathology,” The International Journal of Molecular Sciences 25 (2024): 4199. 10.3390/ijms25084199 PMC11050675 38673785

[110] G. Aubourg , S. J. Rice , P. Bruce‐Wootton , and J. Loughlin , “Genetics of Osteoarthritis,” Osteoarthritis and Cartilage 30 (2022): 636–649. 10.1016/j.joca.2021.03.002 PMC9067452 33722698

[111] J. Li , H. Wang , S. Xu , Y. Liu , and Z. Wang , “Single‐cell RNA Sequencing Unravels Chondrocyte Heterogeneity And Immune Cell Interactions In Knee Osteoarthritis Pathogenesis,” Clinical Rheumatology 45 (2026): 2877–2891. 10.1007/s10067-026-08030-0 41824220

[112] Y. Liu , W. Da , M.‐J. Xu , et al., “Single‐Cell Transcriptomics Reveals Novel Chondrocyte And Osteoblast Subtypes And Their Role In Knee Osteoarthritis Pathogenesis,” Signal Transduction Targeted Therapy 10 (2025): 40. 10.1038/s41392-025-02136-8 PMC11794573 39904988

[113] P. R. Coryell , B. O. Diekman , and R. F. Loeser , “Mechanisms and Therapeutic Implications Of Cellular Senescence In Osteoarthritis,” Nature Reviews Rheumatology 17 (2021): 47–57. 10.1038/s41584-020-00533-7 PMC8035495 33208917

[114] X. Li , L. Fang , R. Zhou , et al., “Current Cutting‐Edge Omics Techniques On Musculoskeletal Tissues And Diseases,” Bone Research 13 (2025): 59. 10.1038/s41413-025-00442-z PMC12146411 40484858

[115] F. Meng , J. He , X. Zhang , et al., “Histone Lactylation Antagonizes Senescence and Skeletal Muscle Aging by Modulating Aging‐Related Pathways,” Advanced Science 12 (2025): 2412747. 10.1002/advs.202412747 PMC12165025 40388671

[116] F. Saberi Hosnijeh , S. M. Bierma‐Zeinstra , and A. C. Bay‐Jensen , “Osteoarthritis year in review,” Osteoarthritis and Cartilage 27 (2018): 412–423. 10.1016/j.joca.2018.12.002 30552966

[117] A. Mobasheri , C. S. Thudium , A.‐C. Bay‐Jensen , et al., “Biomarkers for Osteoarthritis: Current Status And Future Prospects,” Best Pract Res Clin Rheumatol 37 (2023): 101852. 10.1016/j.berh.2023.101852 37620236

[118] Y.‐L. Wu , S.‐G. Wan , Y. Long , et al., “Correlation Between Circulating Cell‐Free Mitochondrial DNA Content And Severity Of Knee Degeneration In Patients With Knee Osteoarthritis: A Cross‐Sectional Study,” Arthritis Research & Therapy 26 (2024): 202. 10.1186/s13075-024-03438-y PMC11571657 39558418

[119] S.‐H. Park , M.‐A. Kang , Y. J. Moon , K. Y. Jang , and J. R. Kim , “Metformin Coordinates Osteoblast/Osteoclast Differentiation Associated with Ischemic Osteonecrosis,” Aging (Albany NY) 12 (2020): 4727–4741. 10.18632/aging.102796 PMC7138543 32045366

[120] A. J. Covarrubias , R. Perrone , A. Grozio , and E. Verdin , “NAD(+) Metabolism And Its Roles In Cellular Processes During Ageing,” Nature Reviews Molecular Cell Biology 22 (2021): 119–141. 10.1038/s41580-020-00313-x PMC7963035 33353981

[121] Y. Zhang , C. Yin , X. Yuan , and S. Xu , “Lactate Exacerbated Rheumatoid Arthritis by Activating the cGAS‐STING Signaling Pathway In Macrophages,” International Immunopharmacology 169 (2026): 115993. 10.1016/j.intimp.2025.115993 41389663

[122] S. M. Haag , M. F. Gulen , L. Reymond , et al., “Targeting STING with Covalent Small‐Molecule Inhibitors,” Nature 559 (2018): 269–273. 10.1038/s41586-018-0287-8 29973723

[123] W. Bao , S. Liu , C. Du , et al., “Energy‐Replenishing, Mitochondria‐Targeted Hydrogel Microspheres Mitigate Sarcopenia Via Cellular Senescence Amelioration,” J Control Release 391 (2026): 114595. 10.1016/j.jconrel.2025.114595 41512967

[124] D. S. C. Han and Y. M. D. Lo , “The Nexus of cfDNA and Nuclease Biology,” Trends Genetics 37 (2021): 758–770. 10.1016/j.tig.2021.04.005 34006390

[125] O. H. Jeon , C. Kim , R.‐M. Laberge , et al., “Local Clearance Of Senescent Cells Attenuates The Development Of Post‐Traumatic Osteoarthritis And Creates A Pro‐Regenerative Environment,” Nature Medicine 23 (2017): 775–781. 10.1038/nm.4324 PMC5785239 28436958

[126] J. N. Farr , M. Xu , M. M. Weivoda , et al., “Targeting Cellular Senescence Prevents Age‐Related Bone Loss In Mice,” Nature Medicine 23 (2017): 1072–1079. 10.1038/nm.4385 PMC5657592 28825716

[127] J. Zhao , L. Zheng , G. Dai , et al., “Senolytics Cocktail Dasatinib And Quercetin Alleviate Chondrocyte Senescence And Facet Joint Osteoarthritis In Mice,” Spine J 25 (2025): 184–198. 10.1016/j.spinee.2024.09.017 39343238

[128] M. P. Murphy and R. C. Hartley , “Mitochondria as a Therapeutic Target For Common Pathologies,” Nature Reviews Drug Discovery 17 (2018): 865–886. 10.1038/nrd.2018.174 30393373

[129] R. Iorio , S. Petricca , V. Mattei , and S. Delle Monache , “Horizontal Mitochondrial Transfer As A Novel Bioenergetic Tool For Mesenchymal Stromal/Stem Cells: Molecular Mechanisms And Therapeutic Potential In A Variety Of Diseases,” Journal of translational medicine 22 (2024): 491. 10.1186/s12967-024-05047-4 PMC11127344 38790026

[130] G. B. Kubat , P. Picone , E. Tuncay , et al., “Biotechnological Approaches And Therapeutic Potential Of Mitochondria Transfer And Transplantation,” Nature Communication 16 (2025): 5709. 10.1038/s41467-025-61239-6 PMC12217326 40593725

[131] J. D. Chavez , X. Tang , M. D. Campbell , et al., “Mitochondrial protein interaction landscape of SS‐31,” The Proceedings of the National Academy of Sciences 117 (2020): 15363–15373. 10.1073/pnas.2002250117 PMC7334473 32554501

[132] J. Kim , H.‐S. Kim , and J. H. Chung , “Molecular Mechanisms of Mitochondrial DNA Release and Activation of the cGAS‐STING Pathway,” Experimental & Molecular Medicine 55 (2023): 510–519. 10.1038/s12276-023-00965-7 PMC10037406 36964253

[133] L. M. Lasko , C. G. Jakob , R. P. Edalji , et al., “Discovery of a Selective Catalytic p300/CBP Inhibitor that Targets Lineage‐Specific Tumours,” Nature 550 (2017): 128–132. 10.1038/nature24028 PMC6050590 28953875

[134] E. A. Bordt , P. Clerc , B. A. Roelofs , et al., “The Putative Drp1 Inhibitor mdivi‐1 Is a Reversible Mitochondrial Complex I Inhibitor that Modulates Reactive Oxygen Species,” Developmental Cell 40 (2017): 583–594.e6. 10.1016/j.devcel.2017.02.020 PMC5398851 28350990

[135] M. Békés , D. R. Langley , and C. M. Crews , “PROTAC Targeted Protein Degraders: The Past Is Prologue,” Nature Reviews Drug Discovery 21 (2022): 181–200. 10.1038/s41573-021-00371-6 PMC8765495 35042991

[136] M. S. Gadd , A. Testa , X. Lucas , et al., “Structural basis of PROTAC Cooperative Recognition For Selective Protein Degradation,” Nature Chemical Biology 13 (2017): 514–521. 10.1038/nchembio.2329 PMC5392356 28288108

[137] W. Zhou , G. Sun , Z. Zhang , et al., “Proteasome‐Independent Protein Knockdown by Small‐Molecule Inhibitor for the Undruggable Lung Adenocarcinoma,” Journal of the American Chemical Society 141 (2019): 18492–18499. 10.1021/jacs.9b08777 31657561

[138] D. Wang , W. Wang , L. Fang , et al., “Mitochondrial Protease Targeting Chimeras for Mitochondrial Matrix Protein Degradation,” Journal of the American Chemical Society 145 (2023): 12861–12869. 10.1021/jacs.3c03756 37276358

[139] B. Y. Mok , M. H. de Moraes , J. Zeng , et al., “A Bacterial Cytidine Deaminase Toxin Enables CRISPR‐Free Mitochondrial Base Editing,” Nature 583 (2020): 631–637. 10.1038/s41586-020-2477-4 PMC7381381 32641830

[140] S.‐I. Cho , K. Lim , S. Hong , et al., “Engineering TALE‐Linked Deaminases To Facilitate Precision Adenine Base Editing In Mitochondrial DNA,” Cell 187 (2024): 95–109.e26. 10.1016/j.cell.2023.11.035 38181745

[141] K. Kong , B. Li , Y. Chang , et al., “Delivery of FGF18 using mRNA‐LNP Protects The Cartilage Against Degeneration Via Alleviating Chondrocyte Senescence,” J Nanobiotechnology 23 (2025): 34. 10.1186/s12951-025-03103-9 PMC11753171 39844298

[142] X. Zhao , J. Lin , M. Liu , et al., “Targeting FAP‐Positive Chondrocytes in Osteoarthritis: A Novel Lipid Nanoparticle siRNA Approach to Mitigate Cartilage Degeneration,” J Nanobiotechnology 22 (2024): 659. 10.1186/s12951-024-02946-y PMC11515236 39456041

[143] M. V. Berridge , R. T. Schneider , and M. J. McConnell , “Mitochondrial Transfer from Astrocytes to Neurons following Ischemic Insult: Guilt by Association?,” Cell Metabolism 24 (2016): 376–378. 10.1016/j.cmet.2016.08.023 27626198

[144] T. Ahmad , S. Mukherjee , B. Pattnaik , et al., “Miro1 Regulates Intercellular Mitochondrial Transport & Enhances Mesenchymal Stem Cell Rescue Efficacy,” EMBO Journal 33 (2014): 994–1010. 10.1002/embj.201386030 PMC4193933 24431222

[145] S. Nie , Y. Yu , R. Yan , et al., “Bone‐Targeted Mitochondrial Delivery Via Magnetic‐Temperature Responsive Artificial Cells For Repairing Age‐Related Fractures,” Frontiers in Pharmacology 16 (2026): 2025. 10.3389/fphar.2025.1725973 PMC12808401 41552830

[146] E. Peveri , T. Sganga , E. Peveri , et al., “From Preservation To Repair: Mitochondrial Transplantation As A Paradigm Shift In Organ Transplantation,” Hepatobiliary & Pancreatic Diseases International 25 (2025): 306–314. 10.1016/j.hbpd.2025.10.003 41193376

[147] M. Zhang , W. Hu , C. Cai , Y. Wu , J. Li , and S. Dong , “Advanced Application Of Stimuli‐Responsive Drug Delivery System For Inflammatory Arthritis Treatment,” Material Today Biology 14 (2022): 100223. 10.1016/j.mtbio.2022.100223 PMC8881671 35243298

[148] J. Cao , W. Lu , Y. Lv , et al., “Mitochondria‐Targeted Nanocarriers For Smart Response Delivery Of Natural Products: A review,” J Drug Target 34 (2026): 1116–1132. 10.1080/1061186X.2025.2611940 41504123

[149] R. Zhou , W. Fu , D. Vasylyev , S. G. Waxman , and C.‐J. Liu , “Ion Channels In Osteoarthritis: Emerging Roles And Potential Targets,” Nature Reviews Rheumatology 20 (2024): 545–564. 10.1038/s41584-024-01146-0 PMC12374755 39122910

[150] W. Sun , S. Chi , Y. Li , et al., “The Mechanosensitive Piezo1 Channel Is Required For Bone Formation,” Elife 8 (2019): 47454. 10.7554/eLife.47454 PMC6685704 31290742

[151] T. Jiang , F. Yu , Y. Zhou , et al., “Synergistic Effect Of Ultrasound And Reinforced Electrical Environment By Bioinspired Periosteum For Enhanced Osteogenesis Via Immunomodulation Of Macrophage Polarization Through Piezo1,” Material Today Biology 27 (2024): 101147. 10.1016/j.mtbio.2024.101147 PMC11263955 39045313

[152] H. H. Szeto , “First‐In‐Class Cardiolipin‐Protective Compound As A Therapeutic Agent To Restore Mitochondrial Bioenergetics,” The British Journal of Pharmacology 171 (2014): 2029–2050. 10.1111/bph.12461 PMC3976620 24117165

[153] M. Suda , K. H. Paul , U. Tripathi , T. Minamino , T. Tchkonia , and J. L. Kirkland , “Targeting Cell Senescence and Senolytics: Novel Interventions for Age‐Related Endocrine Dysfunction,” Endocr Rev 45 (2024): 655–675. 10.1210/endrev/bnae010 PMC11405506 38500373

[154] E. J. Topol , “High‐Performance Medicine: The Convergence Of Human And Artificial Intelligence,” Nature Medicine 25 (2019): 44–56. 10.1038/s41591-018-0300-7 30617339

[155] S. Duan , Q. Zhang , J. Zhu , and J. Wang , “SS‐31 Targets NOS2 to Enhance Osteogenic Differentiation in Aged BMSCs by Restoring Mitochondrial Function,” Organogenesis 21 (2025): 2519649. 10.1080/15476278.2025.2519649 PMC12258809 40570323

[156] D. H. Mendelsohn , N. Walter , W.‐H. Cheung , et al., “Targeting Mitochondria In Bone And Cartilage Diseases: A Narrative Review,” Redox Biology 83 (2025): 103667. 10.1016/j.redox.2025.103667 PMC12136924 40354767

[157] T. J. de Vries , A. S. Kleemann , J. Jin , et al., “The Differential Effect of Metformin on Osteocytes, Osteoblasts, and Osteoclasts,” Current Osteoporosis Reports 21 (2023): 743–749. 10.1007/s11914-023-00828-0 PMC10724308 37796390

[158] K. Zhang , T. Wang , G.‐F. Sun , et al., “Metformin Protects Against Retinal Ischemia/Reperfusion Injury Through AMPK‐Mediated Mitochondrial Fusion,” Free Radic Biol Med 205 (2023): 47–61. 10.1016/j.freeradbiomed.2023.05.019 37253410

[159] F. Pan , Y. Wang , Y. Z. Lim , et al., “Metformin for Knee Osteoarthritis in Patients With Overweight or Obesity: A Randomized Clinical Trial,” JAMA, J Am Med Assoc 333 (2025): 1804–1812. 10.1001/jama.2025.3471 PMC12022862 40274279

[160] K. H. Park‐Min , “Metabolic Reprogramming in Osteoclasts,” Semin Immunopathol 41 (2019): 565–572. 10.1007/s00281-019-00757-0 PMC7671717 31552471

[161] J. N. Justice , A. M. Nambiar , T. Tchkonia , et al., “Senolytics in Idiopathic Pulmonary Fibrosis: Results From A First‐In‐Human, Open‐Label, Pilot Study,” EBioMedicine 40 (2019): 554–563. 10.1016/j.ebiom.2018.12.052 PMC6412088 30616998

[162] N. Lane , B. Hsu , J. Visich , B. Xie , A. Khan , and J. Dananberg , “A phase 2, Randomized, Double‐Blind, Placebo‐Controlled Study Of Senolytic Molecule UBX0101 in the Treatment Of Painful Knee Osteoarthritis,” Osteoarthritis and Cartilage 29 (2021): S52–S53.

[163] M. Yoshino , J. Yoshino , B. D. Kayser , et al., “Nicotinamide Mononucleotide Increases Muscle Insulin Sensitivity In Prediabetic Women,” Science 372 (2021): 1224–1229. 10.1126/science.abe9985 PMC8550608 33888596

[164] K. F. Mills , S. Yoshida , L. R. Stein , et al., “Long‐Term Administration of Nicotinamide Mononucleotide Mitigates Age‐Associated Physiological Decline in Mice,” Cell Metabolism 24 (2016): 795–806. 10.1016/j.cmet.2016.09.013 PMC5668137 28068222

[165] M. J. Rossman , J. R. Santos‐Parker , C. A. C. Steward , et al., “Chronic Supplementation With a Mitochondrial Antioxidant (MitoQ) Improves Vascular Function in Healthy Older Adults,” Hypertension 71 (2018): 1056–1063. 10.1161/HYPERTENSIONAHA.117.10787 PMC5945293 29661838

[166] J. N. Katz , K. R. Arant , and R. F. Loeser , “Diagnosis and Treatment of Hip and Knee Osteoarthritis: A Review,” JAMA 325 (2021): 568–578. 10.1001/jama.2020.22171 PMC8225295 33560326

[167] P. Maudens , O. Jordan , and E. Allémann , “Recent Advances In Intra‐Articular Drug Delivery Systems For Osteoarthritis Therapy,” Drug Discov Today 23 (2018): 1761–1775. 10.1016/j.drudis.2018.05.023 29792929

[168] C. D. DiDomenico , M. Lintz , and L. J. Bonassar , “Molecular Transport In Articular Cartilage — What Have We Learned From The Past 50 years?,” Nature Reviews Rheumatology 14 (2018): 393–403. 10.1038/s41584-018-0033-5 29899547

[169] F. Ordikhani , N. Zandi , M. Mazaheri , et al., “Targeted Nanomedicines For The Treatment Of Bone Disease And Regeneration,” Med Res Rev 41 (2021): 1221–1254. 10.1002/med.21759 33347711

[170] M. He , C. Cao , Z. Ni , et al., “PROTACs: Great Opportunities For Academia And Industry (an update from),” Signal Transduction Targeted Therapy 7 (2022): 181. 10.1038/s41392-022-00999-9 PMC9178337 35680848

[171] A. Kalinkovich and G. Livshits , “The cross‐talk between the cGAS‐STING Signaling Pathway And Chronic Inflammation In The Development Of Musculoskeletal Disorders,” Ageing Research Reviews 104 (2025): 102602. 10.1016/j.arr.2024.102602 39612990

[172] Z. Han , K. Wang , S. Ding , and M. Zhang , “Cross‐talk of Inflammation And Cellular Senescence: A New Insight Into The Occurrence And Progression Of Osteoarthritis,” Bone Research 12 (2024): 69. 10.1038/s41413-024-00375-z PMC11615234 39627227

[173] Y. Wu , H. Hu , T. Wang , W. Guo , S. Zhao , and R. Wei , “Characterizing Mitochondrial Features In Osteoarthritis Through Integrative Multi‐Omics And Machine Learning Analysis,” Front Immunol 15 (2024): 1414301. 10.3389/fimmu.2024.1414301 PMC11254675 39026663

[174] S. Castagno , M. Birch , M. van der Schaar , and A. McCaskie , “Predicting Rapid Progression In Knee Osteoarthritis: A Novel And Interpretable Automated Machine Learning Approach, With Specific Focus On Young Patients And Early Disease,” Annals of the Rheumatic Diseases 84 (2025): 124–135. 10.1136/ard-2024-225872 39874226

[175] G. S. Gulati , J. P. D'Silva , Y. Liu , L. Wang , and A. M. Newman , “Profiling Cell Identity And Tissue Architecture With Single‐Cell And Spatial Transcriptomics,” Nature Reviews Molecular Cell Biology 26 (2025): 11–31. 10.1038/s41580-024-00768-2 39169166

[176] L. A. Low , C. Mummery , B. R. Berridge , C. P. Austin , and D. A. Tagle , “Organs‐on‐chips: Into the Next Decade,” Nature Reviews Drug Discovery 20 (2021): 345–361. 10.1038/s41573-020-0079-3 32913334

[177] D. E. Ingber , “Human Organs‐On‐Chips For Disease Modelling, Drug Development And Personalized Medicine,” Nature Reviews Genetics 23 (2022): 467–491. 10.1038/s41576-022-00466-9 PMC8951665 35338360

[178] P. Occhetta , A. Mainardi , E. Votta , et al., “Hyperphysiological Compression Of Articular Cartilage Induces An Osteoarthritic Phenotype In A Cartilage‐On‐A‐Chip Model,” Nature Biomedical Engineering 3 (2019): 545–557. 10.1038/s41551-019-0406-3 31160722

[179] C. A. Paggi , L. M. Teixeira , S. Le Gac , and M. Karperien , “Joint‐On‐Chip Platforms: Entering A New Era Of In Vitro Models For Arthritis,” Nature Reviews Rheumatology 18 (2022): 217–231. 10.1038/s41584-021-00736-6 35058618

[180] Y. S. Zhang , J. Aleman , S. R. Shin , et al., “Multisensor‐Integrated Organs‐On‐Chips Platform For Automated And Continual In Situ Monitoring Of Organoid Behaviors,” The Proceedings of the National Academy of Sciences 114 (2017): E2293–E2302. 10.1073/pnas.1612906114 PMC5373350 28265064

[181] R. Laubenbacher , B. Mehrad , I. Shmulevich , and N. Trayanova , “Digital Twins in Medicine,” Nat Comput Sci 4 (2024): 184–191. 10.1038/s43588-024-00607-6 PMC11102043 38532133

[182] K. Zhang , X. Yang , Y. Wang , et al., “Artificial Intelligence in Drug Development,” Nature Medicine 31 (2025): 45–59. 10.1038/s41591-024-03434-4 39833407

[183] J. N. Acosta , G. J. Falcone , P. Rajpurkar , and E. J. Topol , “Multimodal Biomedical AI,” Nature Medicine 28 (2022): 1773–1784. 10.1038/s41591-022-01981-2 36109635

[184] M. Zhang , T. Zhang , R. Zou , et al., “Mitochondrial Quality Control As A Therapeutic Target In Cardiovascular Disease: Mechanistic Insights And Future Directions,” Journal of Translational Internal Medicine 13 (2025): 211–240. 10.1515/jtim-2025-0030 PMC12392085 40896290

[185] M. H. Porteus , “A New Class of Medicines through DNA Editing,” New England Journal of Medicine 380 (2019): 947–959. 10.1056/NEJMra1800729 30855744

[186] A. Heikkinen , L. Uusitalo‐Kylmälä , I. Blom , et al., “The Divergent Effects of Nicotinamide Riboside and High‐Intensity Exercise Training on Skeletal Muscle Epigenetic Aging,” Aging cell 25 (2026): e70638. 10.1111/acel.70638 PMC13388492 42481916

[187] M. Shirley , “Elamipretide: First Approval,” Drugs 86 (2026): 377–383. 10.1007/s40265-025-02269-8 41335372

